# Evidence, not eminence, for surgical management during COVID-19: a multifaceted systematic review and a model for rapid clinical change

**DOI:** 10.1093/bjsopen/zrab048

**Published:** 2021-08-05

**Authors:** J G Kovoor, D R Tivey, C D Ovenden, W J Babidge, G J Maddern

**Affiliations:** 1 University of Adelaide, Adelaide, South Australia, Australia; 2 Australian Safety and Efficacy Register of New Interventional Procedures–Surgical, Royal Australasian College of Surgeons, Adelaide, South Australia, Australia; 3 Research, Audit and Academic Surgery, Royal Australasian College of Surgeons, Adelaide, South Australia, Australia; 4 Discipline of Surgery, The Queen Elizabeth Hospital, University of Adelaide, Adelaide, South Australia, Australia

## Abstract

**Background:**

Coronavirus (COVID-19) forced surgical evolution worldwide. The extent to which national evidence-based recommendations, produced by the current authors early in 2020, remain valid, is unclear. To inform global surgical management and a model for rapid clinical change, this study aimed to characterize surgical evolution following COVID-19 through a multifaceted systematic review.

**Methods:**

Rapid reviews were conducted targeting intraoperative safety, personal protective equipment and triage, alongside a conventional systematic review identifying evidence-based guidance for surgical management. Targeted searches of PubMed and Embase from 31 December 2019 were repeated weekly until 7 August 2020, and systematic searches repeated monthly until 30 June 2020. Literature was stratified using Evans’ hierarchy of evidence. Narrative data were analysed for consistency with earlier recommendations. The systematic review rated quality using the AGREE II and AMSTAR tools, was registered with PROSPERO, CRD42020205845. Meta-analysis was not conducted.

**Results:**

From 174 targeted searches and six systematic searches, 1256 studies were identified for the rapid reviews and 21 for the conventional systematic review. Of studies within the rapid reviews, 903 (71.9 per cent) had lower-quality design, with 402 (32.0 per cent) being opinion-based. Quality of studies in the systematic review ranged from low to moderate. Consistency with recommendations made previously by the present authors was observed despite 1017 relevant subsequent publications.

**Conclusion:**

The evidence-based recommendations produced early in 2020 remained valid despite many subsequent publications. Weaker studies predominated and few guidelines were evidence-based. Extracted clinical solutions were globally implementable. An evidence-based model for rapid clinical change is provided that may benefit surgical management during this pandemic and future times of urgency.

## Teaser

From 174 targeted searches and six systematic searches, 1,256 studies were identified for the rapid reviews and 21 for the conventional systematic review; of studies within the rapid reviews, 903 (71.9%) had lower-quality design, with 402 (32%) being opinion-based, quality in the systematic review ranged from low to moderate. Our April 2020 evidence-based recommendations for surgical practice during COVID-19 remained clinically valid despite 1,017 relevant subsequent publications, and extracted clinical solutions were globally implementable. An evidence-based model for rapid clinical change is provided that may benefit surgical management during the COVID-19 era and future times of urgency.

## Introduction

Coronavirus (COVID-19) has challenged surgical practice worldwide^1^, forcing staff to adapt and departments to restructure^2^. Surgery on patients infected with severe acute respiratory syndrome coronavirus 2 (SARS-CoV-2) produces poor outcomes^3^^,^^4^ making appropriate preoperative screening a necessity^5^. Transmission capability^6^ and biodistribution^7^ of the virus threaten the safety of operating room staff, especially those exposed to surgical smoke and aerosol-generating procedures^8^^,^^9^. Disruption associated with the pandemic may burden surgical systems for close to a year^10^, despite vaccination at population level and adaptations in management to cope with SARS-CoV-2 mutations.

Ideological differences in both operative and non-operative delivery of care existing within the global surgery community^11^ have increased since COVID-19. This heterogeneity was reflected in the early operative recommendations, based mainly on expert opinions, and left considerable uncertainty^12^^,^^13^. Past consensus collaborations with a common philosophy have improved surgical safety worldwide^14^^,^^15^, and there is need for similar efforts to maintain effective surgical care during the COVID-19 era^16^.

Evidence-based principles have previously guided surgical innovation at an international level^17^. In April 2020, observing the need for rapid clinical change in response to COVID-19, the authors used this philosophy to produce guidance for the urgent adaptation of surgical services on a national scale^18^. Targeted rapid reviews were combined with the advice of clinical experts to produce evidence-based recommendations for three aspects of practice: safety of intraoperative practice for open *versus* laparoscopic surgery^19^^,^^20^, use of personal protective equipment (PPE)^21^^,^^22^ and surgical triage^23^^,^^24^. As time has passed and the COVID-19 literature has grown^25^, it is unclear if these recommendations remain valid.

Although reliable models exist for clinical change in surgical management^17^, none achieve this in the rapid fashion required for urgent circumstances. As the COVID-19 pandemic has forced a uniquely rapid series of modifications to surgical services worldwide, understanding changes in the surgical literature since the identification of SARS-CoV-2^26^ may benefit the development of a model for rapid evidence-based adaptations of surgical services.

To inform surgical practice during the COVID-19 era with an evidence-based model for rapid clinical change in surgical management, a multifaceted systematic review was conducted. This aimed to characterize the evolution of surgery since the identification of SARS-CoV-2, through rapid reviews targeting three aspects of surgical care (intraoperative safety^19^^,^^20^, PPE^21^^,^^22^ and surgical triage^23^^,^^24^) complementing a conventional systematic review of published evidence-based guidance for surgical practice during the pandemic.

## Methods

The rapid data sharing that followed the COVID-19 outbreak^25^ prompted a multifaceted approach to systematic review^1^. All searches were staggered temporally to identify subtle updates to the evidence base. Both rapid review and conventional systematic review methodologies were utilized in a complementary fashion within a multifaceted approach. The same rapid review methodology used during April 2020^19–24^^,^^27^ was implemented to characterize the evolution in the surgical literature during the pandemic. Searches targeting the three aforementioned areas of interest^19–24^ were repeated at approximately weekly intervals. In contrast, conventional systematic review methodology was repeated at monthly intervals to evaluate the quality and evolution of published guidance for surgery derived from a formal literature search.

No language or publication restrictions were applied. The research questions and inclusion criteria were established *a priori*. As some articles were published in preprint or ‘in press’ form in addition to their final publication format^25^, searches were date-restricted by date of database entry rather than date of publication. Duplicates were removed when identified, with the earliest record retained. Searches were supplemented by consultation of current reviews and original research relating to surgery during the COVID-19 pandemic identified through targeted searches of PubMed and Google Scholar.

### Rapid reviews

Searches sought to identify studies of any design, in any setting, directly relevant to three surgical topic areas: safety of intraoperative practice (including endoscopy procedures of the gastrointestinal or respiratory tract)^19^^,^^20^, use of PPE^21^^,^^22^ and surgical triage^23^^,^^24^. Due to the similarity in PPE requirements for surgical and non-surgical staff, studies relevant to PPE in non-surgical settings were also sought. Six search strings were developed targeting safe intraoperative practice^19^^,^^20^, PPE^21^^,^^22^ and surgical triage^23^^,^^24^ in PubMed (incorporating MEDLINE) and Embase. These six searches were retrospectively conducted four times per month at approximately weekly intervals from 31 December 2019 (identification of SARS-CoV-2)^26^ to 7 August 2020, producing 174 searches over 29 time points across this time frame.

A single reviewer screened titles and abstracts for relevance to the three dilemmas^19–24^, and reviewed full texts of relevant articles to extract data using a standard extraction form. Where full texts could not be obtained, data extraction was performed using the study abstract if possible. Screening results and data extraction were regularly cross-checked by three reviewers at intervals of 7–14 days between May and August 2020. Disagreements were resolved by consensus, with one reviewer acting as arbitrator if required. Data were extracted for study design, major themes and statements that updated the surgical literature from the rapid reviews developed in April 2020^19–24^. Extracted data from studies with design ranking above ‘poor’ for all dimensions within Evans’ hierarchy of evidence^28^, or those providing novel data to the surgical literature, were favoured in data synthesis. Following data extraction and thematic analysis of extracted narrative data, four reviewers inspected the authors’ initial evidence-based recommendations (developed in April 2020)^19–24^ for accuracy as of August 2020.

Data were synthesized in narrative and tabular formats. Outcomes of interest were: the proportion of relevant studies of weaker or stronger design according to Evans’ hierarchy of evidence^28^, proportion of each study design, major themes within the narrative data of the relevant studies, and consistency of narrative data in August 2020 with the April 2020 evidence-based recommendations^19–24^.

### Conventional systematic review

Conventional systematic review was conducted according to Preferred Reporting Items for Systematic Reviews and Meta-Analyses (PRISMA) guidelines^29^. The study protocol was prospectively registered on PROSPERO (CRD42020205845). Studies were eligible for inclusion if they provided or summarized recommendations at the specialty level or more broadly for surgical practice during the COVID-19 pandemic, and outlined a search strategy in the article or abstract that identified at least one database or search term. Studies of any design, in any setting, were searched for within PubMed (incorporating MEDLINE) and Embase using the search terms (surgery* OR surgical* OR surgeon*) AND (‘COVID-19’ OR coronavirus OR ‘SARS-CoV-2’ OR ‘2019-nCoV’ OR ‘corona virus’ OR ‘COVID’). This search was repeated six times, at monthly intervals, between 31 December 2019 and 30 June 2020.

Two reviewers independently screened titles and abstracts, reviewed full texts and extracted data using a standard extraction form. Screening of titles and abstracts was facilitated through use of a web application (Rayyan, Qatar Computing Research Institute, Ar-Rayyan, Qatar)^30^. Disagreements were resolved by consensus, with a third reviewer acting as arbitrator if required. Data were extracted for study design, setting, surgical specialty or area, methodological quality information, major themes, and statements providing recommendations for surgical practice during COVID-19.

Data were synthesized from all included studies in both narrative and tabular formats. The search did not identify any original research eligible for inclusion, so the likelihood of publication bias could not be assessed. The primary outcome was methodological quality of the included studies, which was independently assessed by two reviewers using the Assessment of Multiple Systematic Reviews (AMSTAR) tool^31^ for included systematic reviews, and the Appraisal of Guidelines, Research and Evaluation (AGREE II) tool^32^ for all other included studies. The secondary outcome was dissemination of data within the peer-reviewed literature, which was inferred from individual numbers of citations according to targeted searches of Scopus (until 27 August 2020). For any studies unable to be identified on Scopus, numbers of citations were obtained through targeted searches of Google Scholar (until 27 August 2020). Four reviewers evaluated thematic evolution in extracted narrative data. Meta-analysis was not conducted due to heterogeneity within the included studies.

## Results

**Fig. 1:**
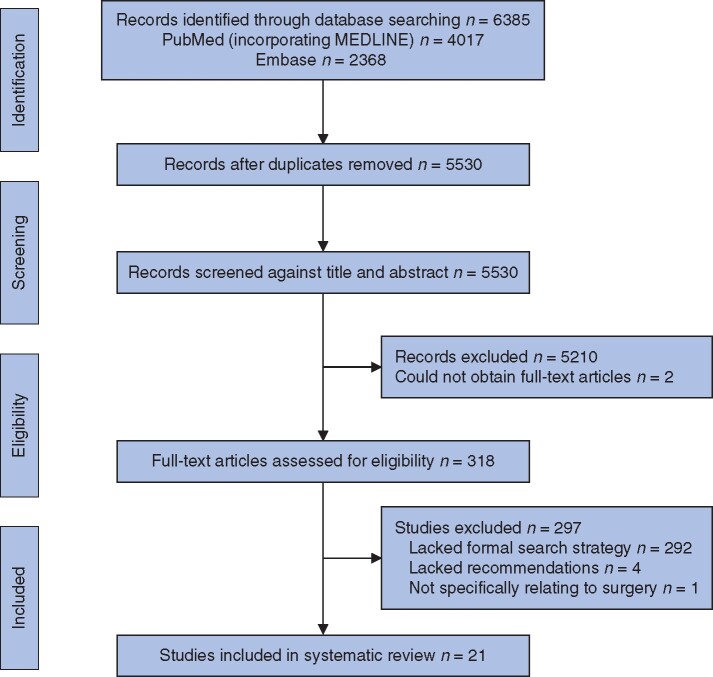
Overall study selection for the conventional systematic review within this study

Via 174 targeted searches staggered at approximately weekly intervals over 29 time points between 31 December 2019 and 7 August 2020, a total of 5238 records (4116 unique reports) were identified, of which 1256 were included in the rapid reviews (*Table S1*). The six systematic searches, at six time points at monthly intervals between 31 December 2019 and 30 June 2020, identified a total of 6385 records (5530 unique reports). From this total, 318 full-text articles were retrieved, of which 21 studies were included in the systematic review (*Fig. 1* and *Fig**s S1–**S**6*). Two full texts could not be obtained for evaluation, despite eligibility being identified on screening of title and abstract^33^^,^^34^.

### Rapid reviews

**Fig. 2:**
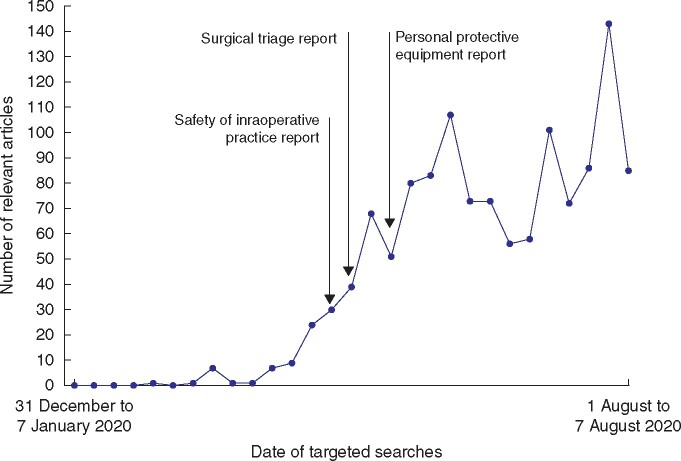
Weekly volume of relevant articles included in rapid reviews

The weekly volume of studies relevant to the surgical areas of interest^19–24^ increased with time during the pandemic, peaking between 22 July and 31 July, when 143 studies were retrieved (*Fig. 2*). After the publication of the authors’ initial recommendations^20^^,^^22^^,^^24^, 1017 relevant articles were retrieved. Of the relevant studies within the rapid reviews, 903 (72 per cent) had a lower-quality design (*Fig.**S7*)^28^, with 402 (32 per cent) being opinion-based evidence or letters (*Table 1*).

**Table 1:** Composition of body of evidence from 1256 records included in the rapid reviews

Study design	**Records** (*n* = 1256)
Lower-quality studies*	903 (71.9)
Opinion-based evidence and letters	402 (32.0)
Expert consensus recommendations	209 (16.6)
Narrative review or recommendations	186 (14.8)
Case reports	17 (1.4)
Descriptive or methodology studies	68 (5.4)
Simulation studies, including cadaver or animal models	21 (1.7)
**Higher-quality studies^†^**	353 (28.1)
Observational studies	43 (3.4)
Cross-sectional survey studies	86 (6.8)
Systematic reviews	37 (2.9)
Evidence-based guidance or recommendations	48 (3.8)
Randomized controlled trials	2 (0.2)
Prospective audits	8 (0.6)
Scoping reviews	41 (3.3)
Rapid reviews	3 (0.2)
Retrospective data analyses	78 (6.2)
Case series	6 (0.5)
Case-control studies	1 (0.1)

Values in parentheses are percentages.

* Lower-quality evidence ranked ‘poor’ for at least one dimension within Evans’ hierarchy of evidence^28^.

† Higher-quality evidence ranked above ‘poor’ for all dimensions within Evans’ hierarchy of evidence^28^.

**Fig. 3:**
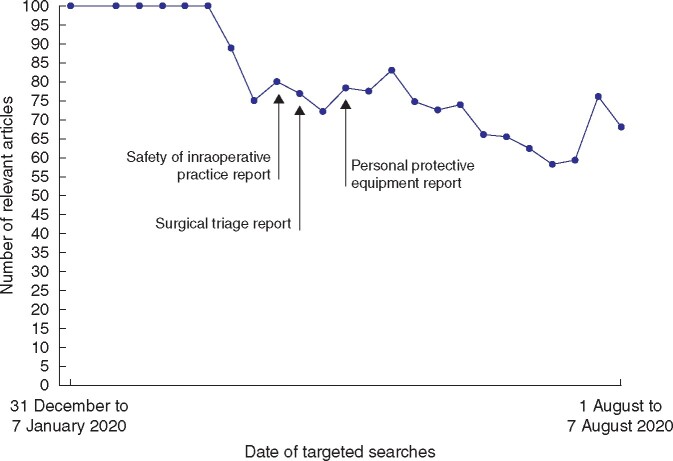
Weekly proportion of lower-quality evidence within the surgical literature during the COVID-19 pandemic

The majority of relevant studies were of lower quality throughout the 7-month time frame (median 77 (i.q.r. 71–92) per cent for searches retrieving at least one relevant study), although the proportion reduced in the latter weeks (*Fig. 3*). Opinion-based evidence and letters formed the largest proportion of relevant studies (median 32 (i.q.r. 18–40) per cent), predominating in all months apart from March and April (*Table S2*, *Fig**s S8* and *S9*). Expert consensus recommendations were the most frequent study design between 1 March and 30 April (median 72 (i.q.r. 44–100) per cent for March, median 32 (i.q.r. 25–39) per cent for April), and formed a median of 16 (i.q.r. 11–28) per cent for the overall time frame (*Table S2* and *Fig.**S10*).

The first relevant study, chronologically, classified as stronger evidence was an observational study identified in the final search of March 2020^35^. Observational studies were more prevalent later in the time frame, particularly in June 2020 (median 6 (i.q.r. 5–7) per cent), peaking in the final week of analysis with 11 per cent (*Table S2* and *Fig.**S11*). Retrospective data analyses were the most common forms of stronger evidence (median 4 (i.q.r. 0–7) per cent) (*Table 1*, *Fig.**S12*). However, these were only retrieved after 15 April, peaking between 1 July and 31 July 2020 (median 8 (i.q.r. 7–11) per cent) (*Table S2* and *Fig.**S13*).

From the 174 targeted searches, 48 (4 per cent) evidence-based recommendations were retrieved (median 2 (i.q.r. 0–4) per cent), compared with 209 (17 per cent) expert consensus recommendations in the same time frame (median 16 (i.q.r. 11–28) per cent) (*Table**s**S1* and *S2*). The earliest evidence-based recommendations were retrieved in the first week of April 2020. Expert consensus recommendations were retrieved at rates ranging from two to 13 times that of evidence-based recommendations between 1 April and 31 July 2020 (*Fig**s S14* and *S15*).

### Conventional systematic review

The conventional systematic review identified 21 studies across six continents and various surgical specialties (*Table 2*). Three studies (14 per cent) were retrieved in April^12^^,^^36^^,^^37^, nine (43 per cent) in May^16^^,^^38–45^ and nine (43 per cent) in June 2020^46–54^. Regarding study type, eleven (52 per cent) were scoping reviews^12^^,^^16^^,^^37^^,^^38^^,^^40^^,^^44^^,^^46^^,^^48–50^^,^^53^, eight (38 per cent) systematic reviews^36^^,^^39^^,^^41^^,^^42^^,^^45^^,^^47^^,^^51^^,^^54^ and two (10 per cent) were narrative reviews that fulfilled the inclusion criteria^43^^,^^52^.

**Table 2:** Characteristics of studies providing or summarizing evidence-based recommendations for surgery during COVID-19 included in systematic review

Study (month, year)	Study design	Region	Surgical specialty or area	Methodological quality		Number of citations*
				AGREE II score (%)	Average AMSTAR score (/11)	
**COVIDSurg Collaborative^12^ (April, 2020)**	Scoping review	International	Surgical care	49	–	126
**Hirschmann *et al.*** ^36^ **(April, 2020)**	Systematic review	International	Orthopaedic and trauma surgery	–	3	21
**Zimmermann and Nkenke^37^ (April, 2020)**	Scoping review	Austria	Oral and maxillofacial surgery	32	–	18
**Boccalatte *et al*.** ^38^ **(May, 2020)**	Scoping review	Argentina	Head and neck, and otolaryngology	27	–	2
**De Simone *et al*.** ^39^ **(May, 2020)**	Systematic review	International	Emergency surgery	–	1	8
**Germano *et al*.** ^40^ **(May, 2020)**	Scoping review	Italy	Neurosurgery	26	–	3
**Hojaij *et al*.** ^41^ **(May, 2020)**	Systematic review	Brazil	Surgical practice	–	3	3
**Moletta *et al*.** ^42^ **(May, 2020)**	Systematic review	Italy	Surgery	–	5	4
**Puliatti *et al*.** ^43^ **(May, 2020)**	Narrative review with database stated	International	Urology	34	–	20
**Soreide *et al*.** ^16^ **(May, 2020)**	Scoping review	International	Surgical services	48	–	85
**Spock *et al*.** ^44^ **(May, 2020)**	Scoping review	USA	Transnasal surgery	46	–	0
**Welsh Surgical Research Initiative Collaborative^45^ (May, 2020)**	Systematic review	UK	Operating theatre practice	–	7·5	1
**Daigle *et al*.^46^ (June, 2020)**	Scoping review	Canada	Oculofacial plastic and orbital surgery	63	–	1
**Heldwein *et al*.** ^47^ ** (June, 2020)**	Systematic review	International	Urology	–	5	0
**Krajewska *et al*.** ^48^ ** (June, 2020)**	Scoping review	Poland	Head and neck, and otolaryngology	49	–	0
**Lagos *et al*.** ^49^ ** (June, 2020)**	Scoping review	Chile	Otolaryngology	47	–	0^**^
**Mintz *et al*.** ^50^ **(June, 2020)**	Scoping review	International	Laparoscopy and laparotomy	42	–	3
**Pini Prato *et al*.** ^51^ ** (June, 2020)**	Systematic review	International	Minimally invasive paediatric surgery	–	3	0
**Shokri *et al*.** ^52^ ** (June, 2020)**	Narrative review with databases stated	USA	Facial plastic and reconstructive surgery	37	–	0
**Viswanathan *et al*.** ^53^ ** (June, 2020)**	Scoping review	India	Spinal surgery	34	–	0^**^
**Wang *et al*.** ^54^ ** (June, 2020)**	Systematic review	International	Orthopaedic surgery	–	4	0

* According to Scopus as of 27 August 2020.

** Unable to be identified on Scopus, thus according to Google Scholar as of 27 August 2020.

Methodological quality scores ranged from low to moderate for the 21 studies (*Table 2*). Of the 13 studies appraised using the AGREE II instrument^12^^,^^16^^,^^37^^,^^38^^,^^40^^,^^43^^,^^44^^,^^46^^,^^48–50^^,^^52^^,^^53^, only one (score 63 per cent)^46^ scored over 50 per cent. Scores of the other 12 studies ranged from 26–49 per cent. Seven (88 per cent) of the included systematic reviews^36^^,^^39^^,^^41^^,^^42^^,^^47^^,^^51^^,^^54^ received average AMSTAR scores below 6 (range 1–5), indicating poor methodological quality. Only one systematic review^54^ received an average AMSTAR score above 6 (7.5), indicating good methodological quality.

Numbers of citations for the 21 studies ranged from 0^44^^,^^47–49^^,^^51–54^ to 126^12^ (median 2 (i.q.r. 0–8). However, there were quantitative discrepancies when each month was grouped, with earlier evidence-based recommendations cited more often. For studies retrieved in April 2020^12^^,^^36^^,^^37^, the median number of citations was 21 (i.q.r. 20–74), compared with 3 (i.q.r. 2–8) for May^16^^,^^38–45^ and 0 (i.q.r. 0–0) for June^46–54^.

### Consistency of surgical literature

Ongoing monitoring of narrative data between 31 December 2019 and 7 August 2020 identified no substantive thematic changes over time (*Table S3*). Despite substantial heterogeneity in study design, narrative data relevant to the three aspects of surgical care^19–24^ consistently concurred with the authors’ recommendations, apart from a small number of low-quality studies recommending an open approach over the laparoscopic alternative to minimize the risk of SARS-CoV-2 transmission to operating room staff^55^. As the pandemic progressed, thematic consistency was observed in all areas despite 1017 relevant publications. After accounting for inherent differences in specialty, there was no significant narrative contradiction in the evidence-based recommendations within the conventional systematic review.

Following consideration of the total body of surgical literature published between 31 December 2019 and 7 August 2020, inspection of the initial evidence-based recommendations made in the authors’ three rapid reviews (developed in April 2020)^19^^,^^21^^,^^23^ revealed no strong evidence subsequently published (as of 7 August 2020) to justify significant change in any recommendation (*Table S4*).

## Discussion

This study has provided a comprehensive characterization of the surgical literature and its temporal evolution during the COVID-19 pandemic. Lower-quality studies predominated, with opinion-based articles and letters the most common communications. Quality did not improve over time despite the increasing number of relevant publications. Clinical recommendations were mostly based on expert consensus, opinion or narrative review. There was a dearth of evidence-based guidance, which ranged from low to moderate methodological quality and for which numbers of citations reflected month of database entry. Evidence-based recommendations developed by the present authors in April 2020 remained clinically valid as of 7 August 2020 despite the large number of relevant publications during this intervening period. Although relevant studies of strong design have been published after the final search was completed^56^, these data have generally concurred with the present study. The initial evidence-based recommendations from April 2020 remain valid.

Declaration of the COVID-19 pandemic^57^ was followed by the proliferation of a large volume of related literature, demonstrating unprecedented responsiveness by peer-reviewed medical journals^25^. However, this did not result in corresponding benefit for clinical practice. Most studies were found to be of lower-quality design, and 4 months of further literature did not highlight significant modification to the present authors’ early-stage, evidence-based recommendations^13^.

Rapid reviews with clearly specified research questions can produce similar clinical conclusions to systematic reviews, albeit with lower certainty^27^. The authors’ April 2020 rapid reviews targeting specific clinical dilemmas^18^ provided reliable and timely evidence-based solutions^19–24^.

The quality of surgical literature has been a topic of past controversy^58^. Although improved through consensus efforts valuing evidence-based principles^17^, the present study reveals that COVID-19 may have caused this issue to resurface. The predominance of expert opinion suggests that the global surgery community may have reverted to traditional eminence-based medicine^59^. Deviating from objective data risks differing clinical approaches being advised, potentially compromising safety^60^. Increasing ideological heterogeneity could confound attempts to achieve collective goals for global surgery^11^. A continued increase in global caseload^61^ implies a substantial challenge may be created for the future.

###  

The present study addressed multiple challenges for surgery during the pandemic^1^ identifying clinical solutions that are relatively inexpensive and implementable on a global scale. Surgery on patients infected with SARS-CoV-2 may cause postoperative morbidity and mortality^3^^,^^4^, and this should be understood for preoperative risk–benefit assessment. Adequate delay after infection should be allowed to ensure safety for patients undergoing elective procedures^62^^,^^63^. Operations on COVID-19 patients should ideally occur in designated theatres with negative-pressure ventilation^12^, and suspected COVID-19 patients should wear a surgical mask during perioperative theatre transport^21^. Appropriate preoperative screening for active SARS-CoV-2 infection using patient history and reverse transcription-polymerase chain reaction is essential^5^. Triage should consider local COVID-19 prevalence and hospital resources^23^, and methods of ensuring a safe resumption of elective surgery despite high prevalence have been described^64^. Although caution was advised for laparoscopic surgery throughout the pandemic, the present study found no evidence that laparoscopy presented a greater risk to theatre staff than open surgery with respect to SARS-CoV-2 transmission. However, given theoretical aerosol transmission risk, capture devices should be used with energy sources producing surgical smoke^8^ and desufflation of pneumoperitoneum should be performed using a suction irrigator system attached to a high-efficiency particulate air filter^19^. Given evidence of multisystem biodistribution^7^, all biological material should be treated as a potential SARS-CoV-2 source. PPE with aerosol precautions (N95 respirator or equivalent)^65^ can provide adequate protection against SARS-CoV-2^66^, and is essential during surgical emergencies, aerosol-generating procedures or when a patient is not confirmed to be COVID-19 negative^21^.

The present study included the use of temporally staggered searches to capture subtle updates in a rapidly evolving evidence base potentially missed by searches at a single time point. Synthesis of a large amount of narrative data provided inexpensive potential clinical solutions. To increase worldwide applicability and reduce the risk of bias, searches were not limited by language^67^ and globally accepted methods were used for classifying strength of evidence^28^, reporting methodology^29^ and risk of bias assessment^31^^,^^32^. Despite these strengths, this study has limitations. The most significant of which was potential bias accompanying the selection of relevant studies for the rapid reviews. The rapid reviews targeted key dilemmas for pandemic surgery^19–24^. In spite of ensuring clinical relevance, this may have limited scope. The paucity of strong evidence in the reviews prompted caution regarding statistical bias within included studies. Heterogeneity in study design prevented meta-analysis, requiring data synthesis in multiple formats for maximal clinical utility and acknowledgement of likely biases.

###  

Characterization of temporal changes in the surgical literature through a multifaceted systematic review confirmed correctness of an evidence-based approach tailored to surgical management during the COVID-19 pandemic. Synthesis of these results will provide surgical staff with an enhanced understanding of the trustworthiness and dissemination of the recommendations within the provided studies, that will allow them to decide whether they should adopt the recommendations for clinical care of surgical patients at their local healthcare institutions. Combination of findings from this study with the authors’ experience during the COVID-19 pandemic provides an evidence-based model for rapid clinical change in surgical management (*Table 3*).

**Table 3:** Evidence-based model for rapid clinical change in surgical management based on data and experience during the COVID-19 pandemic

Time	State of literature	Required action
–	Predominantly reports of similar past phenomena, almost no articles directly relevant to current phenomenon	Acknowledgement of need for rapid clinical change in surgical management
0–2 weeks	Predominantly reports of similar past phenomena, literature relevant to current phenomenon begins to be populated by opinion-based evidence	Working group comprising researchers with literature review experience and clinical experts urgently convened; scoping searches of prior literature regarding similar past phenomena and initial evidence relevant to current phenomenon
∼2 weeks	Small evidence base relevant to current phenomenon predominated by opinion-based articles	Clinical experts assess suitability of prior literature regarding similar past phenomena for contributing to recommendations for current phenomenon, and draft initial guidance based on findings from scoping searches that is not disseminated
∼2–3 weeks	Rapid growth predominated by opinion-based evidence, initial studies of stronger design begin to be published	Rapid reviews targeting clinical dilemmas specific to the current phenomenon. Aim to identify relevant literature and gaps in understanding of current phenomenon
∼3 weeks	Predominantly opinion-based evidence, very few studies of stronger design	Clinical experts develop evidence-based guidance based on findings of rapid reviews targeting clinical dilemmas specific to the current phenomenon
3–4 weeks	Predominantly opinion-based evidence, few studies of stronger design	Reports of rapid reviews containing evidence-based guidance refined within working group
∼4 weeks	Predominantly opinion-based evidence, few studies of stronger design	Reports begin to be rapidly circulated in non-refereed format to surgical staff on a large scale through relevant surgical societies
∼4 weeks–4 months	Rapid growth predominated by opinion-based evidence, however proportion of studies of stronger design increases with time	Rapid review methodology targeting clinical dilemmas repeated weekly; non-refereed reports and evidence-based guidance updated based on findings from regular rapid reviews; reports published in peer-reviewed setting
∼1 year	Unknown	Rapid review methodology targeting clinical dilemmas specific to current phenomenon conducted to confirm clinical validity of evidence-based guidance

The authors hope that these findings provide a crucial lesson for global surgery. Widespread adoption of evidence-based methodology targeting specific clinical dilemmas may facilitate the reorganization of surgical systems in a reliable and unified fashion. This model may be valuable for coordinating surgical responses during the COVID-19 era and future times of urgent need.

## Supplementary Material

zrab048_Supplementary_Data
