# Supplementary material for: Evidence, not eminence, for surgical management during COVID-19: a multifaceted systematic review and a model for rapid clinical change
Source: BJS Open. 2021 Aug 5;5(4):zrab048. doi: 10.1093/bjsopen/zrab048 (PMC8342932; doi:10.1093/bjsopen/zrab048)
Supplement: zrab048_Supplementary_Data [file zrab048_supplementary_data.docx]

**Supplementary data:**

**Evidence, not Eminence, for Surgical Management during COVID-19: A Multifaceted Systematic Review and Model for Rapid Clinical Change**

*Joshua G. Kovoor, David R. Tivey, Christopher D. Ovenden,*

*Wendy J. Babidge, Guy J. Maddern*

**Table S1.** Complete dataset stratified by study design from rapid reviews

**Figure S1.** Study selection for 31 December 2019 to 31 January 2020 within systematic review

**Figure S2.** Study selection for 1 February 2020 to 29 February 2020 within systematic review

**Figure S3.** Study selection from 1 March 2020 to 31 March 2020 within systematic review

**Figure S4.** Study selection from 1 April 2020 to 30 April 2020 within systematic review

**Figure S5.** Study selection from 1 May 2020 to 31 May 2020 within systematic review

**Figure S6.** Study selection from 1 June 2020 to 30 June 2020 within systematic review

**Table S2**. Median composition of relevant studies by month within the surgical literature during COVID-19

**Figure S7.** Proportions of lower- and higher-quality evidence within the surgical literature during COVID-19

**Figure S8.** Weekly proportion of opinion-based evidence and letters within the surgical literature during the COVID-19 pandemic

**Figure S9.** Proportions of respective study designs within the lower-quality evidence in the COVID-19 surgical literature

**Figure S10.** Weekly proportion of recommendations derived from expert consensus within the surgical literature during the COVID-19 pandemic

**Figure S11.** Weekly proportion of observational studies within the surgical literature during the COVID-19 pandemic

**Figure S12.** Proportions of respective study designs within higher-quality evidence in the COVID-19 surgical literature

**Figure S13.** Weekly proportion of retrospective data analyses within the surgical literature during the COVID-19 pandemic

**Figure S14.** Weekly proportion of higher-quality evidence within the surgical literature during the COVID-19 pandemic

**Figure S15.** Weekly proportion of case series, case-controlled studies, case reports, cross-sectional survey studies, descriptive studies, evidence-based guidance, narrative reviews or recommendations, prospective audits, rapid reviews, randomised controlled trials, scoping reviews, simulation studies, and systematic reviews within the surgical literature during the COVID-19 pandemic

**Table S3.** Temporal evolution of key narrative data for surgical practice during COVID-19

**Table S4.** Evaluation of initial evidence-based recommendations (April 2020)

**References**

**Table S1.** **Complete dataset stratified by study design from rapid reviews**

|  | 31 December to 7 January | 8 January to 14 January | 15 January to 21 January | 22 January to 31 January | 1 February to 7 February | 8 February to 14 February | 15 February to 21 February | 22 February to 29 February | 1 March to 7 March | 8 March to 14 March | 15 March to 21 March | 22 March to 31 March | 1 April to 7 April | 8 April to 14 April | 15 April to 21 April | 22 April to 30 April | 1 May to 7 May | 8 May to 14 May | 15 May to 21 May | 22 May to 31 May | 1 June to 7 June | 8 June to 14 June | 15 June to 21 June | 22 June to 30 June | 1 July to 7 July | 8 July to 14 July | 15 July to 21 July | 22 July to 31 July | 1 August to 7 August | Overall totals for whole time frame |
| --- | --- | --- | --- | --- | --- | --- | --- | --- | --- | --- | --- | --- | --- | --- | --- | --- | --- | --- | --- | --- | --- | --- | --- | --- | --- | --- | --- | --- | --- | --- |
| Total articles from searches | 1 | 0 | 0 | 0 | 1 | 3 | 5 | 17 | 14 | 9 | 19 | 46 | 96 | 148 | 184 | 366 | 246 | 261 | 333 | 467 | 332 | 311 | 181 | 188 | 444 | 318 | 278 | 685 | 375 | 5328 |
| PubMed PPE searches | 0 | 0 | 0 | 0 | 0 | 3 | 1 | 4 | 6 | 3 | 2 | 13 | 18 | 27 | 26 | 44 | 35 | 23 | 26 | 36 | 22 | 36 | 13 | 21 | 26 | 17 | 10 | 14 | 12 | 438 |
| PubMed safe intraoperative practice searches | 0 | 0 | 0 | 0 | 0 | 0 | 1 | 7 | 3 | 3 | 7 | 18 | 40 | 46 | 60 | 110 | 80 | 85 | 98 | 95 | 61 | 70 | 72 | 78 | 80 | 59 | 58 | 76 | 53 | 1260 |
| PubMed surgical triage searches | 0 | 0 | 0 | 0 | 1 | 0 | 3 | 5 | 3 | 2 | 8 | 10 | 28 | 51 | 64 | 105 | 53 | 71 | 91 | 65 | 58 | 48 | 43 | 58 | 49 | 37 | 34 | 42 | 31 | 960 |
| Embase PPE searches | 1 | 0 | 0 | 0 | 0 | 0 | 0 | 1 | 2 | 1 | 2 | 4 | 6 | 20 | 22 | 59 | 47 | 50 | 64 | 150 | 116 | 79 | 37 | 8 | 162 | 118 | 82 | 298 | 164 | 1493 |
| Embase safe intraoperative practice searches | 0 | 0 | 0 | 0 | 0 | 0 | 0 | 0 | 0 | 0 | 0 | 0 | 1 | 0 | 2 | 13 | 3 | 9 | 14 | 21 | 17 | 13 | 4 | 9 | 24 | 19 | 27 | 53 | 12 | 241 |
| Embase surgical triage searches | 0 | 0 | 0 | 0 | 0 | 0 | 0 | 0 | 0 | 0 | 0 | 1 | 3 | 4 | 10 | 35 | 28 | 23 | 40 | 100 | 58 | 65 | 12 | 14 | 103 | 68 | 67 | 202 | 103 | 936 |
| Number of duplicates removed | 0 | 0 | 0 | 0 | 0 | 0 | 0 | 4 | 2 | 1 | 4 | 7 | 21 | 34 | 53 | 116 | 76 | 70 | 83 | 109 | 77 | 62 | 44 | 48 | 72 | 60 | 50 | 148 | 71 | 1212 |
| Total remaining after duplicates removed | 1 | 0 | 0 | 0 | 1 | 3 | 5 | 13 | 12 | 8 | 15 | 39 | 75 | 114 | 131 | 250 | 170 | 191 | 250 | 358 | 255 | 249 | 137 | 140 | 372 | 258 | 228 | 537 | 304 | 4116 |
| Total relevant articles | 0 | 0 | 0 | 0 | 1 | 0 | 1 | 7 | 1 | 1 | 7 | 9 | 24 | 30 | 39 | 68 | 51 | 80 | 83 | 107 | 73 | 73 | 56 | 58 | 101 | 72 | 86 | 143 | 85 | 1256 |
| Total lower quality evidence | 0 | 0 | 0 | 0 | 1 | 0 | 1 | 7 | 1 | 1 | 7 | 8 | 18 | 24 | 30 | 49 | 40 | 62 | 69 | 80 | 53 | 54 | 37 | 38 | 63 | 42 | 51 | 109 | 58 | 903 |
| Total higher quality evidence | 0 | 0 | 0 | 0 | 0 | 0 | 0 | 0 | 0 | 0 | 0 | 1 | 6 | 6 | 9 | 19 | 11 | 18 | 14 | 27 | 20 | 19 | 19 | 20 | 38 | 30 | 35 | 34 | 27 | 353 |
| Opinion-based evidence and letters | 0 | 0 | 0 | 0 | 1 | 0 | 1 | 7 | 0 | 0 | 2 | 2 | 0 | 6 | 6 | 12 | 18 | 28 | 40 | 43 | 30 | 25 | 16 | 22 | 30 | 6 | 15 | 54 | 38 | 402 |
| Expert consensus recommendations | 0 | 0 | 0 | 0 | 0 | 0 | 0 | 0 | 1 | 1 | 3 | 4 | 10 | 7 | 15 | 17 | 12 | 13 | 11 | 19 | 8 | 12 | 8 | 8 | 10 | 7 | 12 | 24 | 7 | 209 |
| Narrative review or recommendations | 0 | 0 | 0 | 0 | 0 | 0 | 0 | 0 | 0 | 0 | 2 | 2 | 7 | 8 | 8 | 15 | 7 | 14 | 11 | 12 | 9 | 8 | 9 | 3 | 12 | 16 | 16 | 20 | 7 | 186 |
| Case reports | 0 | 0 | 0 | 0 | 0 | 0 | 0 | 0 | 0 | 0 | 0 | 0 | 0 | 0 | 0 | 2 | 0 | 2 | 1 | 0 | 2 | 1 | 1 | 0 | 0 | 2 | 1 | 4 | 1 | 17 |
| Descriptive or methodology studies | 0 | 0 | 0 | 0 | 0 | 0 | 0 | 0 | 0 | 0 | 0 | 0 | 0 | 3 | 1 | 2 | 3 | 4 | 3 | 4 | 4 | 5 | 3 | 3 | 8 | 11 | 3 | 6 | 5 | 68 |
| Simulation studies including cadaver or animal models | 0 | 0 | 0 | 0 | 0 | 0 | 0 | 0 | 0 | 0 | 0 | 0 | 1 | 0 | 0 | 1 | 0 | 1 | 3 | 2 | 0 | 3 | 0 | 2 | 3 | 0 | 4 | 1 | 0 | 21 |
| Observational studies | 0 | 0 | 0 | 0 | 0 | 0 | 0 | 0 | 0 | 0 | 0 | 1 | 0 | 0 | 0 | 1 | 1 | 1 | 0 | 1 | 5 | 4 | 2 | 5 | 1 | 7 | 1 | 4 | 9 | 43 |
| Cross-sectional survey studies | 0 | 0 | 0 | 0 | 0 | 0 | 0 | 0 | 0 | 0 | 0 | 0 | 1 | 2 | 0 | 2 | 3 | 6 | 5 | 10 | 5 | 4 | 4 | 4 | 8 | 7 | 10 | 7 | 8 | 86 |
| Systematic reviews | 0 | 0 | 0 | 0 | 0 | 0 | 0 | 0 | 0 | 0 | 0 | 0 | 1 | 0 | 1 | 1 | 1 | 2 | 2 | 3 | 2 | 5 | 0 | 2 | 6 | 5 | 1 | 4 | 1 | 37 |
| Evidence-based guidance or recommendations | 0 | 0 | 0 | 0 | 0 | 0 | 0 | 0 | 0 | 0 | 0 | 0 | 3 | 1 | 4 | 7 | 2 | 1 | 0 | 5 | 1 | 1 | 2 | 1 | 6 | 3 | 7 | 4 | 0 | 48 |
| Randomised controlled trials | 0 | 0 | 0 | 0 | 0 | 0 | 0 | 0 | 0 | 0 | 0 | 0 | 1 | 0 | 0 | 0 | 0 | 0 | 0 | 0 | 0 | 0 | 0 | 0 | 0 | 0 | 0 | 0 | 1 | 2 |
| Prospective audits | 0 | 0 | 0 | 0 | 0 | 0 | 0 | 0 | 0 | 0 | 0 | 0 | 0 | 1 | 0 | 2 | 0 | 1 | 0 | 0 | 1 | 0 | 0 | 0 | 0 | 0 | 0 | 1 | 2 | 8 |
| Scoping reviews | 0 | 0 | 0 | 0 | 0 | 0 | 0 | 0 | 0 | 0 | 0 | 0 | 0 | 1 | 2 | 2 | 3 | 2 | 4 | 4 | 1 | 1 | 6 | 2 | 6 | 3 | 1 | 2 | 1 | 41 |
| Rapid reviews | 0 | 0 | 0 | 0 | 0 | 0 | 0 | 0 | 0 | 0 | 0 | 0 | 0 | 1 | 0 | 0 | 0 | 0 | 0 | 0 | 0 | 0 | 1 | 1 | 0 | 0 | 0 | 0 | 0 | 3 |
| Retrospective data analyses | 0 | 0 | 0 | 0 | 0 | 0 | 0 | 0 | 0 | 0 | 0 | 0 | 0 | 0 | 1 | 3 | 1 | 5 | 3 | 4 | 5 | 4 | 4 | 5 | 9 | 5 | 14 | 10 | 5 | 78 |
| Case series | 0 | 0 | 0 | 0 | 0 | 0 | 0 | 0 | 0 | 0 | 0 | 0 | 0 | 0 | 1 | 1 | 0 | 0 | 0 | 0 | 0 | 0 | 0 | 0 | 2 | 0 | 1 | 1 | 0 | 6 |
| Case-control studies | 0 | 0 | 0 | 0 | 0 | 0 | 0 | 0 | 0 | 0 | 0 | 0 | 0 | 0 | 0 | 0 | 0 | 0 | 0 | 0 | 0 | 0 | 0 | 0 | 0 | 0 | 0 | 1 | 0 | 1 |

**Figure S1: Study selection for 31 December 2019 to 31 January 2020 within systematic review**

6 records identified through database searching

2 PubMed (incorporating MEDLINE)

4 Embase

5 records after duplicates removed

5 records screened against title and abstract

0 studies included in systematic review

5 records excluded

**Figure S2: Study selection for 1 February 2020 to 29 February 2020 within systematic review**

40 records identified through database searching

38 PubMed (incorporating MEDLINE)

2 Embase

40 records after duplicates removed

40 records screened against title and abstract

2 full-text articles assessed for eligibility

0 studies included in systematic review

38 records excluded

2 studies excluded

2 lacked formal search strategy

**Figure S3: Study selection from 1 March 2020 to 31 March 2020 within systematic review**

0 studies included in systematic review

12 full-text articles assessed for eligibility

109 records excluded

121 records screened against title and abstract

121 records after duplicates removed

126 records identified through database searching

115 PubMed (incorporating MEDLINE)

11 Embase

12 studies excluded

11 lacked formal search strategy

1 not specifically relating to surgery

**Figure S4: Study selection from 1 April 2020 to 30 April 2020 within systematic review**

3 studies included in systematic review

83 full-text articles assessed for eligibility

954 records excluded

1037 records screened against title and abstract

1037 records after duplicates removed

1237 records identified through database searching

933 PubMed (incorporating MEDLINE)

304 Embase

80 studies excluded

77 lacked formal search strategy

3 lacked recommendations

**Figure S5: Study selection from 1 May 2020 to 31 May 2020 within systematic review**

2756 records identified through database searching

1550 PubMed (incorporating MEDLINE)

1206 Embase

453 records after duplicates removed

2303 records screened against title and abstract

2187 records excluded

116 full-text articles assessed for eligibility

9 studies included in systematic review

107 studies excluded

106 lacked formal search strategy

1 lacked recommendations

**Figure S6: Study selection from 1 June 2020 to 30 June 2020 within systematic review**

2220 records identified through database searching

1379 PubMed (incorporating MEDLINE)

841 Embase

2024 records after duplicates removed

2024 records screened against title and abstract

105 full-text articles assessed for eligibility

96 studies excluded

96 lacked formal search strategy

11 studies included in systematic review

2187 records excluded

2 could not obtain full-text articles^1, 2^

**Table S2: Median composition of relevant studies by month within the surgical literature during COVID-19**

| **Study design** | **Median proportion of relevant studies (IQR)** | | | | | | | | |
| --- | --- | --- | --- | --- | --- | --- | --- | --- | --- |
|  | **31 December 2019 to 31 January 2020** | **1 February to 29 February 2020**** | **1 March to 31 March 2020** | **1 April to 30 April 2020** | **1 May to 31 May 2020** | **1 June to 30 June 2020** | **1 July to 31 July 2020** | **1 August to 7 August 2020*** | **Overall time frame (1 February to 7 August 2020)**** |
| Opinion-based evidence and letters | Zero relevant studies retrieved | 100% (100-100%) | 11.1% (0-23.8%) | 16.5% (11.5-18.2%) | 37.7% (35.2-42.2%) | 36.1% (32.8-38.7%) | 23.6% (15.2-31.7%) | 44.7% | 32.0% (17.6-40.4%) |
| Expert consensus recommendations |  | 0 (0-0) | 72.2% (44.0-100%) | 31.7% (24.6-39.3%) | 17% (15.5-19.2%) | 14.0% (13.1-14.8%) | 11.9% (8.9-14.7%) | 8.2% | 16.3% (10.7-28.4%) |
| Narrative review or recommendations |  | 0 (0-0) | 11.1% (0-23.8%) | 24.4% (21.7-27.3%) | 13.5% (12.7-14.7%) | 11.6% (9.5-13.3%) | 16.3% (13.5-19.5%) | 8.2% | 13.5% (7.5-20.9%) |
| Case reports |  | 0 (0-0) | 0 (0-0) | 0 (0-1.0%) | 1.0% (0-1.5%) | 1.6% (1.0-2.0%) | 2.0% (1.0-2.8%) | 1.2% | 0 (0-1.5%) |
| Descriptive or methodology studies |  | 0 (0-0) | 0 (0-0) | 2.8% (1.9-4.7%) | 4.4% (3.7-5.2%) | 5.4% (5.3-5.8%) | 6.1% (4.0-9.8%) | 5.9% | 3.7% (0-5.6%) |
| Simulation studies, including cadaver or animal models |  | 0 (0-0) | 0 (0-0) | 0.7% (0-2.1%) | 1.6% (0.9-2.3%) | 1.7% (0-3.6%) | 1.8% (0.5-3.4%) | 0 | 0 (0-2.1%) |
| Observational studies |  | 0 (0-0) | 0 (0-0) | 0 (0-0.4%) | 1.1% (0.7-1.4%) | 6.2% (5-7.3%) | 2.0% (1.1-4.5%) | 10.6% | 1.0% (0-3.0%) |
| Cross-sectional survey studies |  | 0 (0-0) | 0 (0-0) | 3.6% (2.2-4.8%) | 6.8% (6.0-8.0%) | 6.9% (6.5-7.0%) | 8.8% (7.2-10.2%) | 9.4% | 5.7% (0-7.2%) |
| Systematic reviews |  | 0 (0-0) | 0 (0-0) | 2.0% (1.1-3.0%) | 2.5% (2.3-2.6%) | 3.1% (2.1-4.3%) | 4.4% (2.4-6.2%) | 1.2% | 1.7% (0-2.8%) |
| Evidence-based guidance or recommendations |  | 0 (0-0) | 0 (0-0) | 10.3% (8.5-10.8%) | 2.6% (0.9-4.1%) | 1.5% (1.4-2.2%) | 5.1% (3.8-6.5%) | 0 | 1.5% (0-4.3%) |
| Randomised controlled trials |  | 0 (0-0) | 0 (0-0) | 0 (0-1.0%) | 0 (0-0) | 0 (0-0) | 0 (0-0) | 1.2% | 0 (0-0) |
| Prospective audits |  | 0 (0-0) | 0 (0-0) | 1.5% (0-3.0%) | 0 (0-0·3%) | 0 (0-0.3%) | 0 (0-0.1%) | 2.4% | 0 (0-0.2%) |
| Scoping reviews |  | 0 (0-0) | 0 (0-0) | 3.1% (2.2-3.8%) | 4.3% (3.4-5.1%) | 2.4% (1.4-5.2%) | 2.8% (1.3-4.6%) | 1.2% | 1.4% (0-3.8%) |
| Rapid reviews |  | 0 (0-0) | 0 (0-0) | 0 (0-0.8%) | 0 (0-0) | 0.9% (0-1.7%) | 0 (0-0) | 0 | 0 (0-0) |
| Retrospective data analyses |  | 0 (0-0) | 0 (0-0) | 1.3% (0-3.0%) | 3.8% (3.2-4.4%) | 7.0% (6.5-7.5%) | 8.0% (7.0-10.8%) | 5.9% | 3.7% (0-6.9%) |
| Case series |  | 0 (0-0) | 0 (0-0) | 0.7% (0-1.7%) | 0 (0-0) | 0 (0-0) | 0.9% (0.5-1.4%) | 0 | 0 (0-0) |
| Case-control studies |  | 0 (0-0) | 0 (0-0) | 0 (0-0) | 0 (0-0) | 0 (0-0) | 0 (0-0.2%) | 0 | 0 (0-0) |

*Raw proportion presented; insufficient data to provide median or IQR

**8 February to 14 February 2020 omitted as zero relevant studies retrieved

**
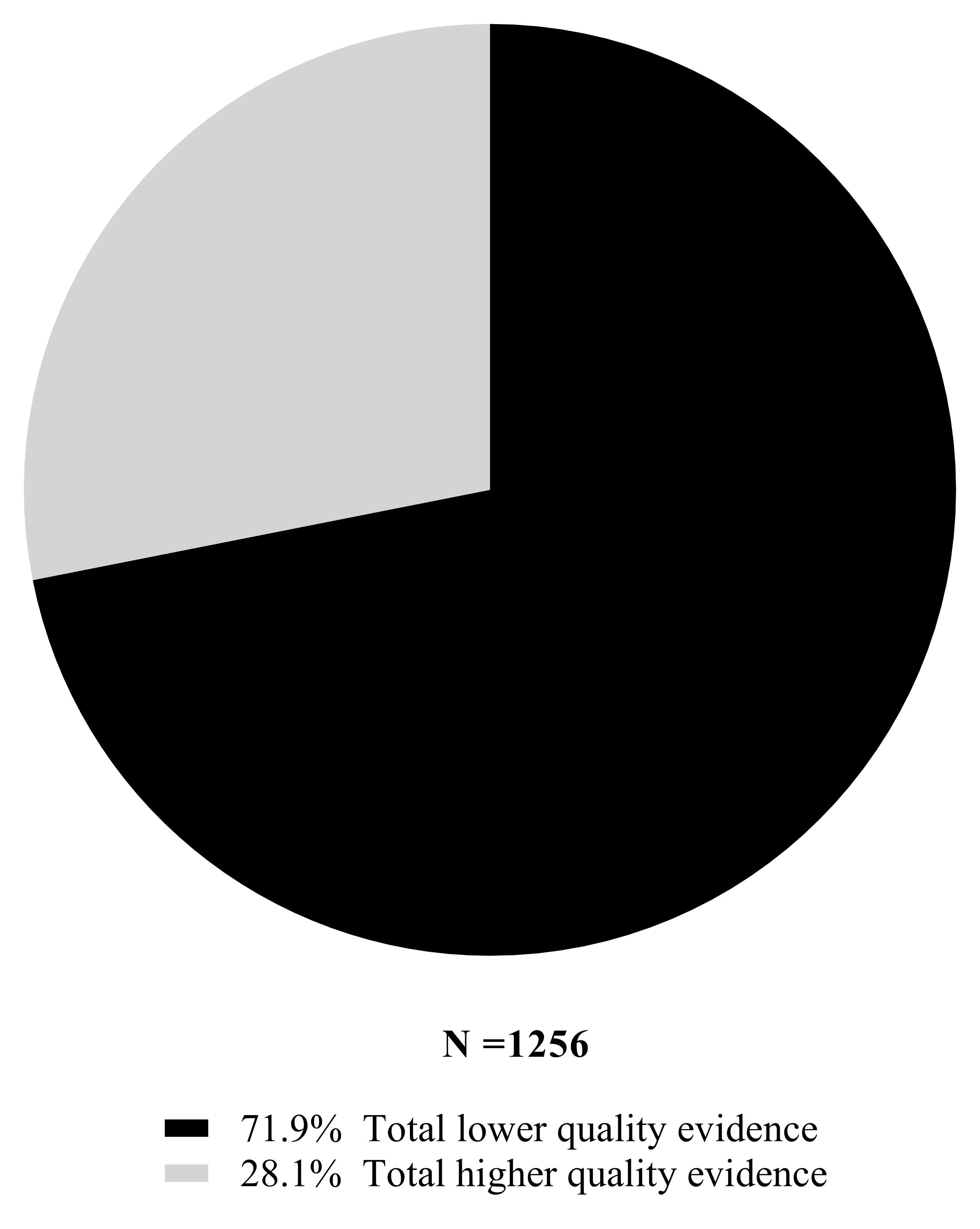
**

**Figure S7: Proportions of lower- and higher-quality evidence within the surgical literature during COVID-19**

Notes: lower-quality evidence ranked ‘poor’ for at least one dimension within Evans’ hierarchy of evidence.^4^ Higher-quality evidence ranked above ‘poor’ for all dimensions within Evans’ hierarchy of evidence.^4^


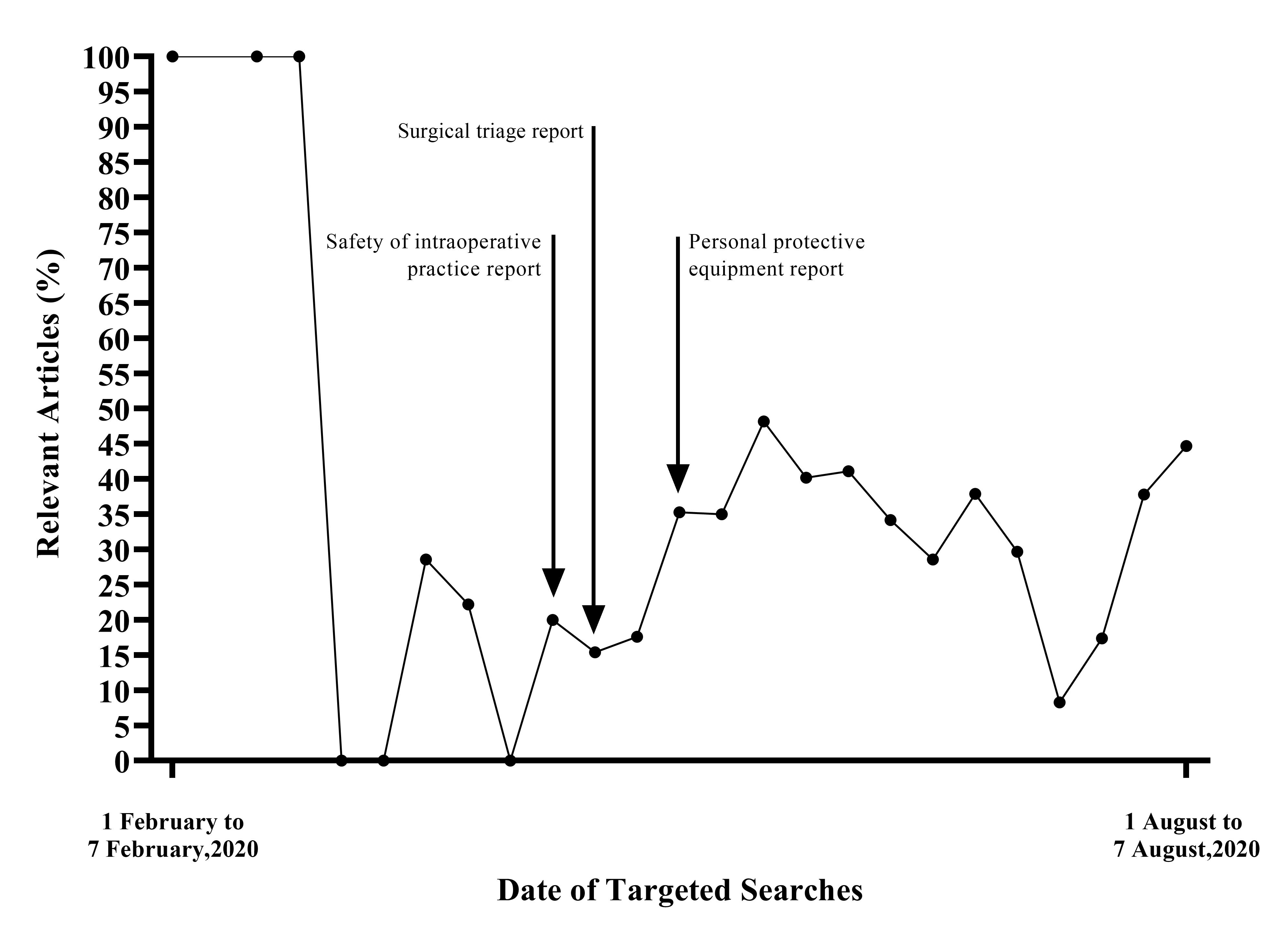


**Figure S8: Weekly proportion of opinion-based evidence and letters within the surgical literature during the COVID-19 pandemic**


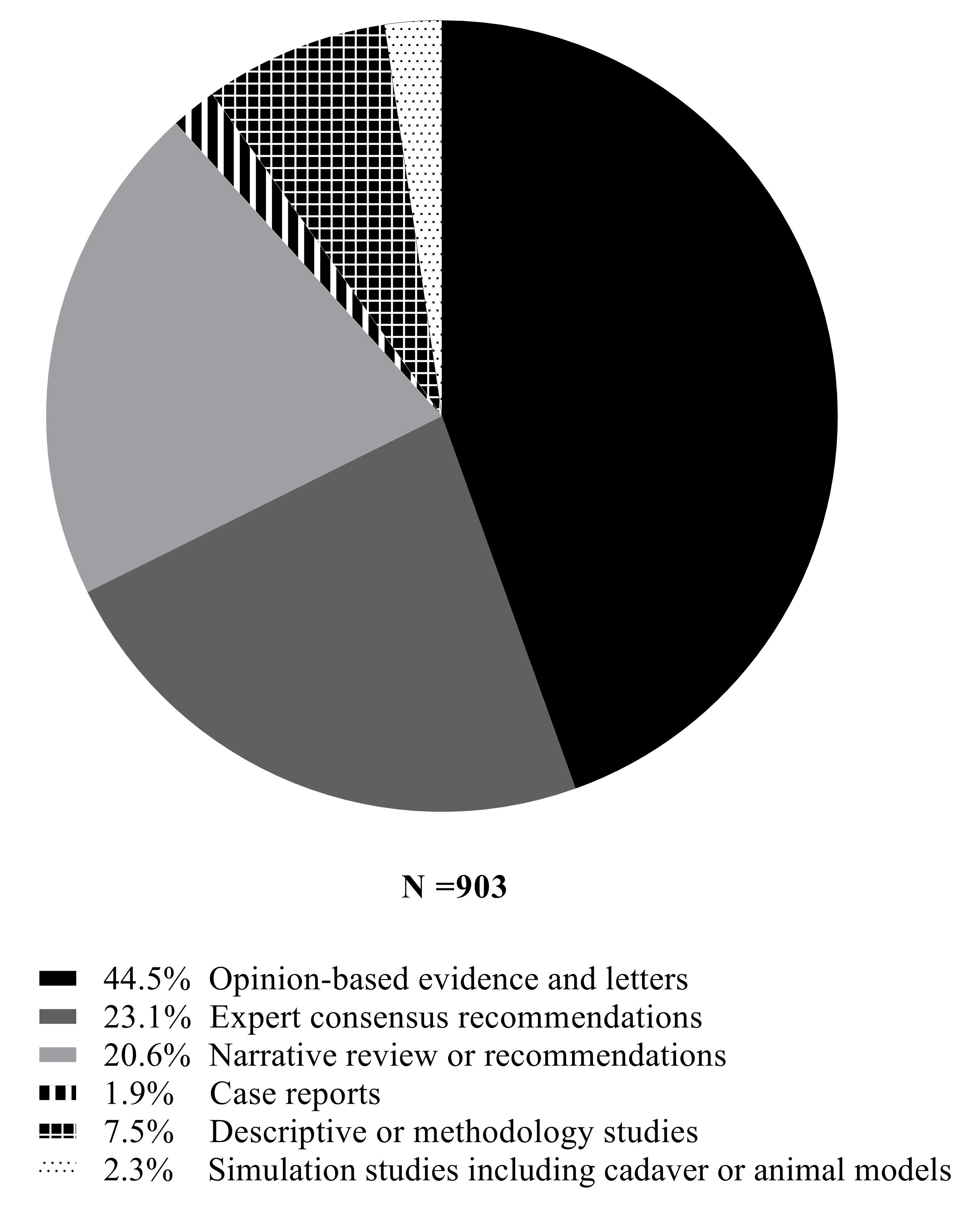


**Figure S9: Proportions of respective study designs within the lower-quality evidence in the COVID-19 surgical literature**


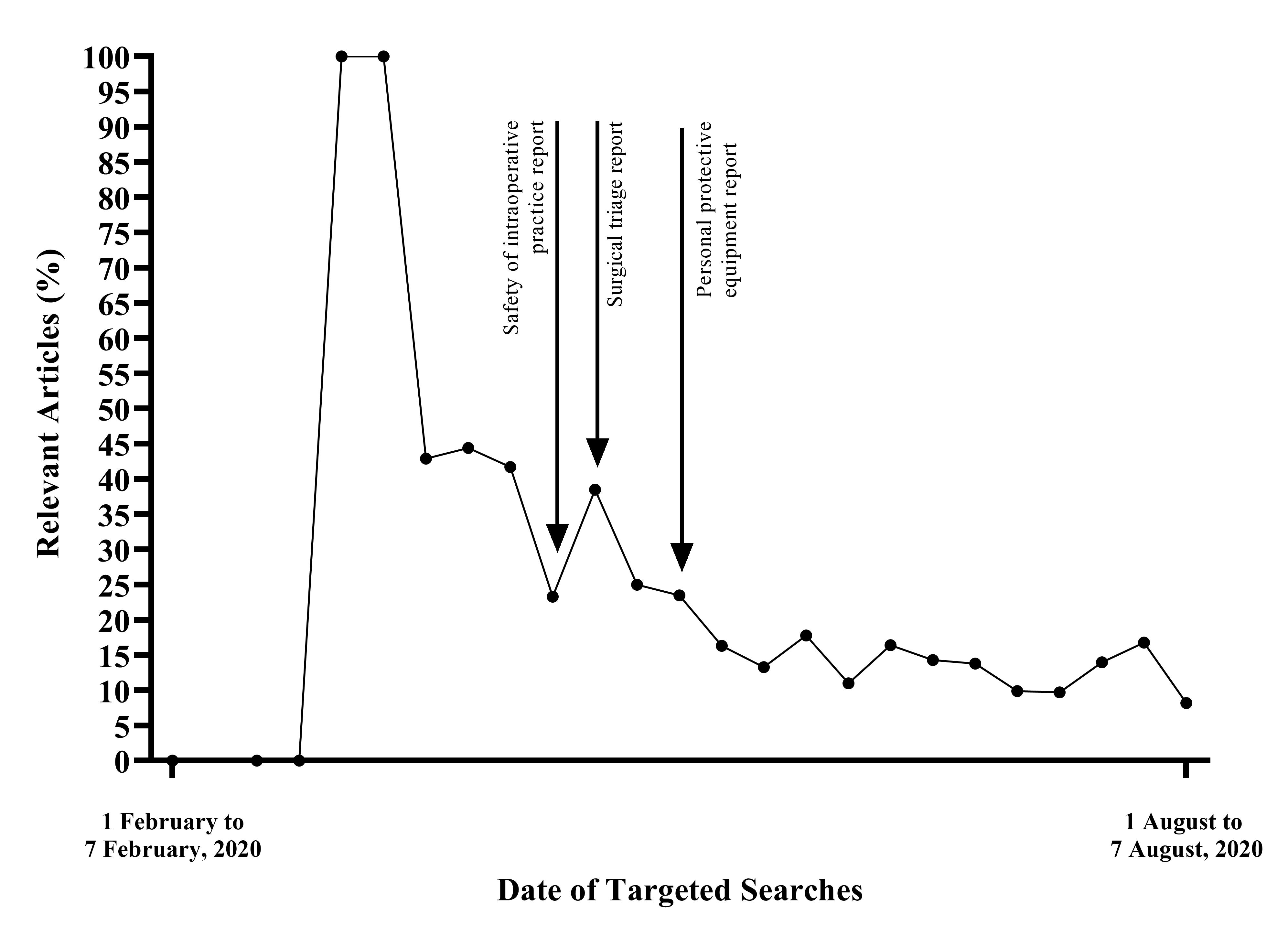


**Figure S10: Weekly proportion of recommendations derived from expert consensus within the surgical literature during the COVID-19 pandemic**


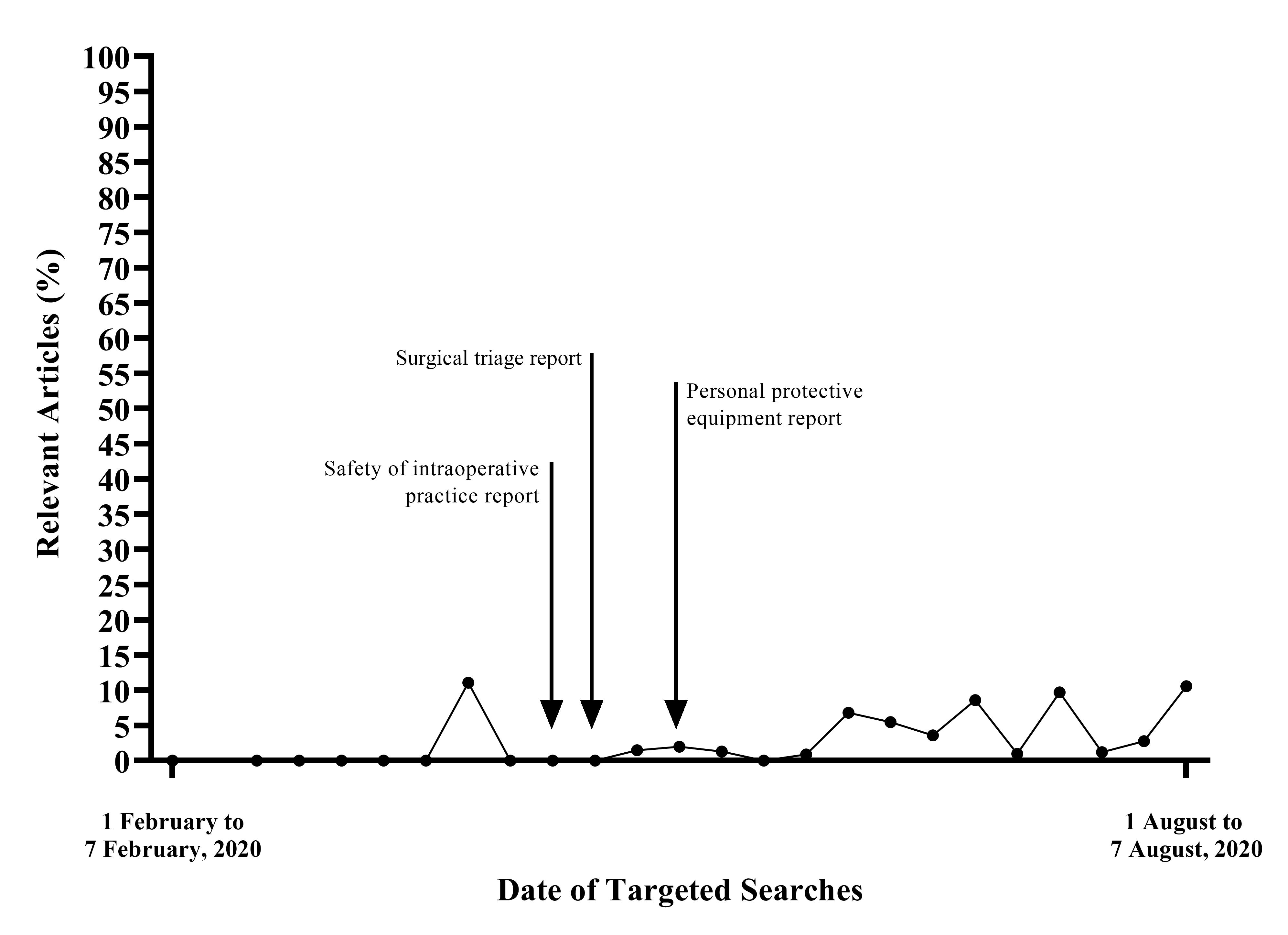


**Figure S11: Weekly proportion of observational studies within the surgical literature during the COVID-19 pandemic**


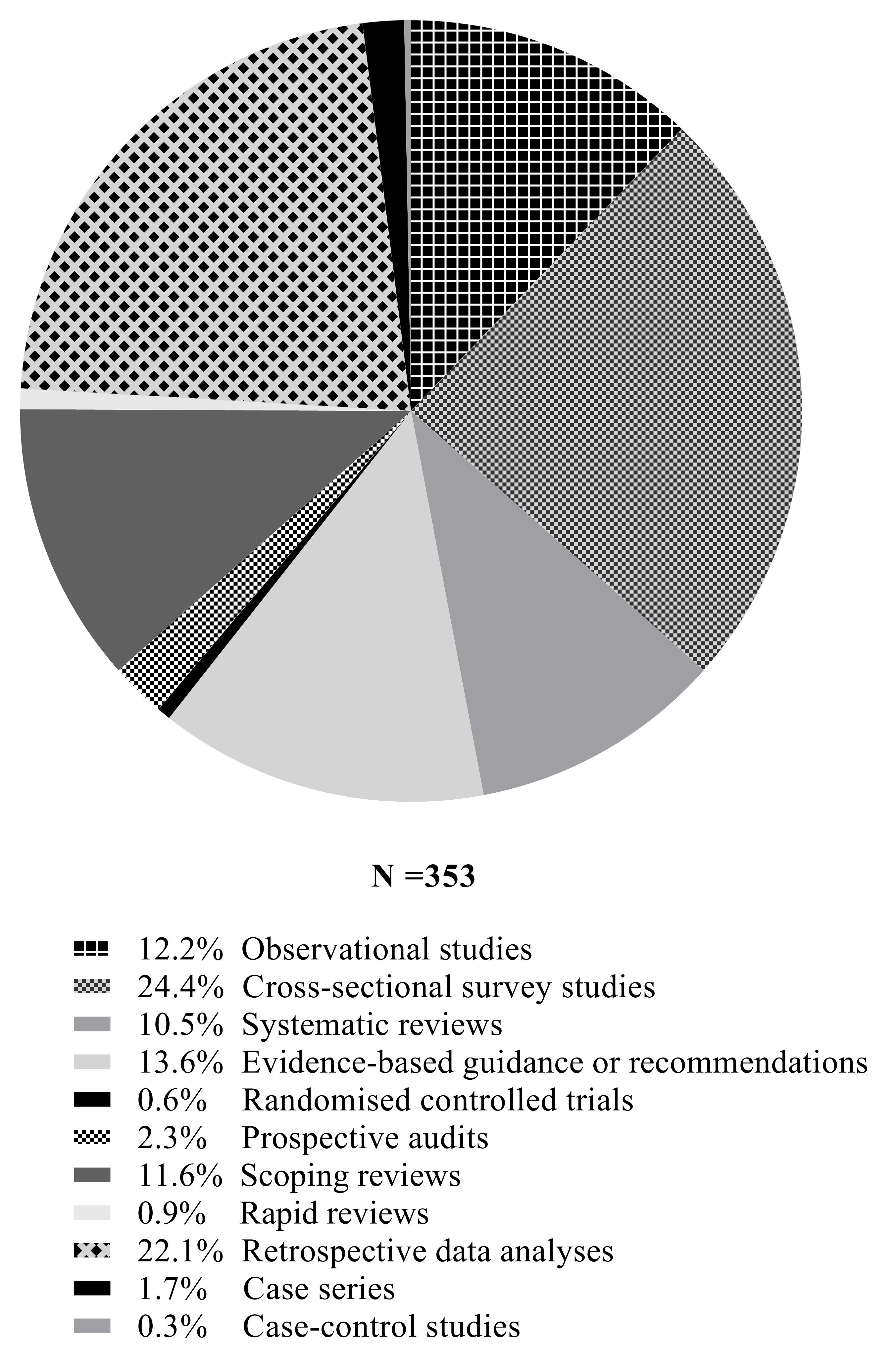


**Figure S12: Proportions of respective study designs within higher-quality evidence in the COVID-19 surgical literature**


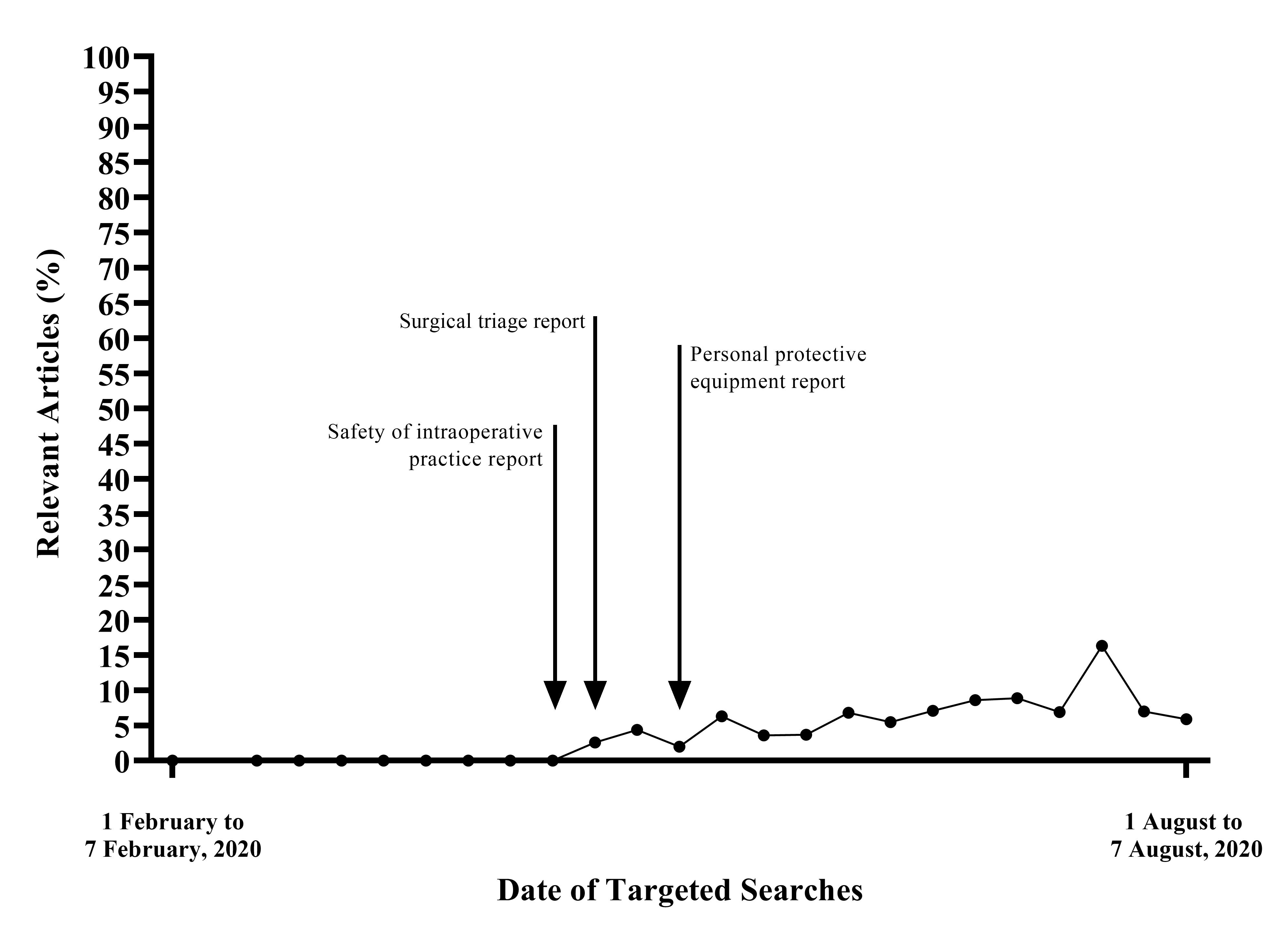


**Figure S13: Weekly proportion of retrospective data analyses within the surgical literature during the COVID-19 pandemic**

**
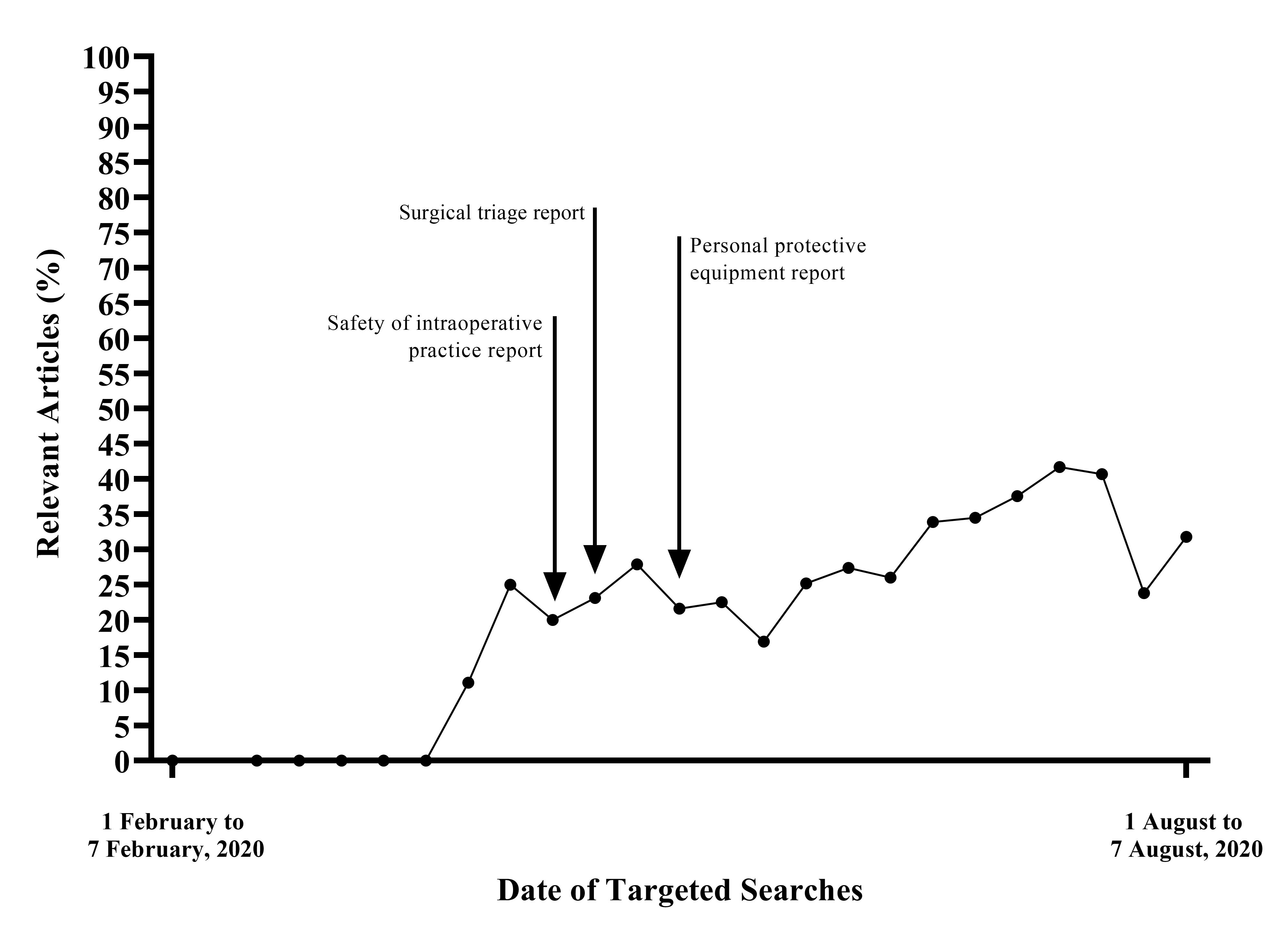
**

**Figure S14: Weekly proportion of higher-quality evidence within the surgical literature during the COVID-19 pandemic**

**
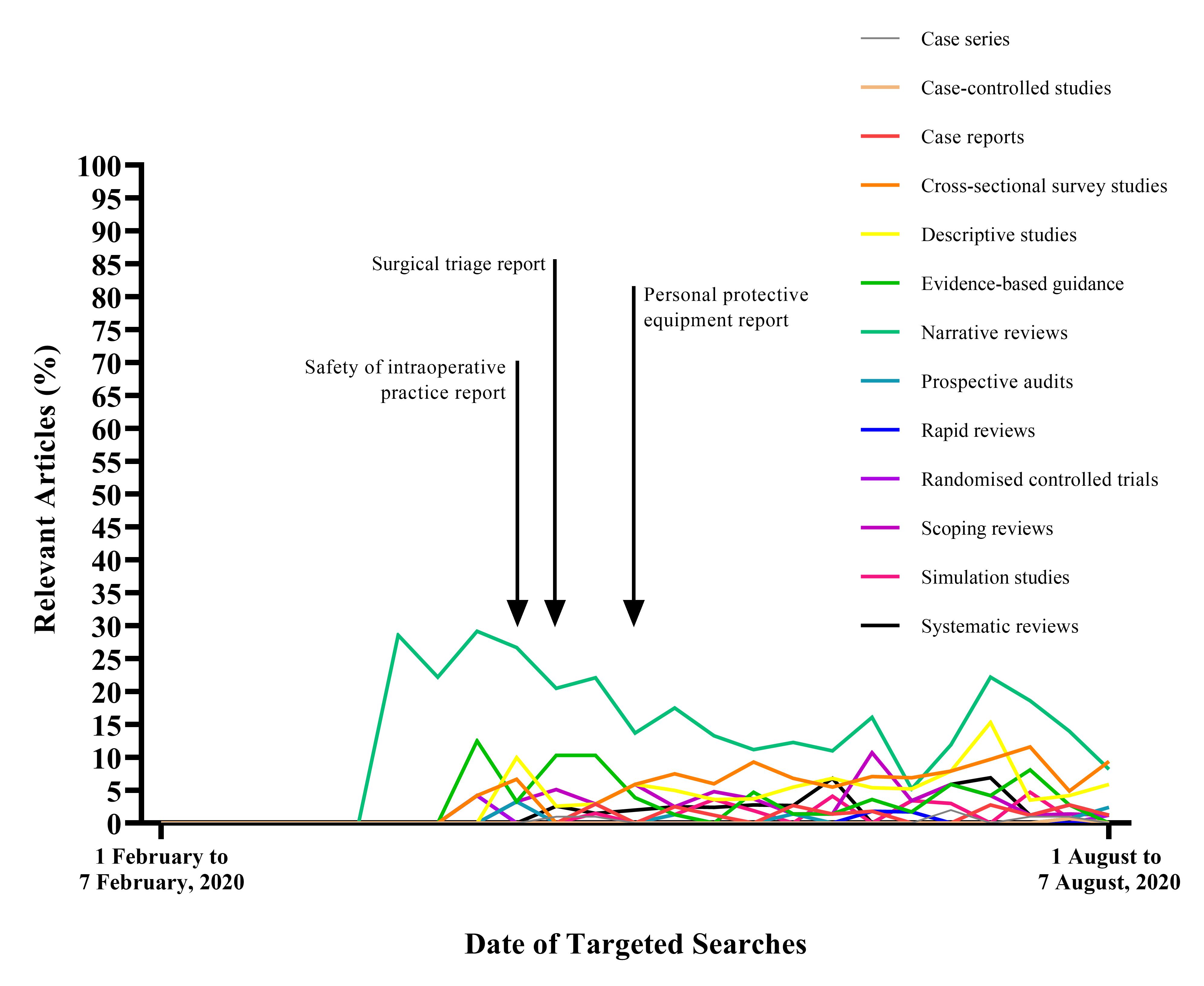
**

**Figure S15: Weekly proportion of case series, case-controlled studies, case reports, cross-sectional survey studies, descriptive studies, evidence-based guidance, narrative reviews or recommendations, prospective audits, rapid reviews, randomised controlled trials, scoping reviews, simulation studies, and systematic reviews within the surgical literature during the COVID-19 pandemic**

**Table S3: Temporal evolution of key narrative data for surgical practice during COVID-19**

| **Timeframe*** | **Intraoperative practice**** | **PPE**** | **Surgical triage**** | **Evidence-based guidance for surgery***** |
| --- | --- | --- | --- | --- |
| 31 December 2019**** to 7 January 2020 | No relevant studies identified from targeted searches | No relevant studies identified from targeted searches | No relevant studies identified from targeted searches | No relevant studies identified from systematic searches |
| 8 January 2020 to 14 January 2020 |  |  |  |  |
| 15 January 2020 to 21 January 2020 |  |  |  |  |
| 22 January 2020 to 31 January 2020 |  |  |  |  |
| 1 February 2020 to 7 February 2020 |  |  |  |  |
| 8 February 2020 to 14 February 2020 |  |  |  |  |
| 15 February 2020 to 21 February 2020 |  |  |  |  |
| 22 February 2020 to 29 February 2020 | Aerosols should be strictly managed in laparoscopic surgery^5^ |  | Operations on benign masses in the gastrointestinal tract should be postponed^6^ |  |
| 1 March 2020 to 7 March 2020 | Micro-aerosol generating ophthalmology procedures should be postponed and nasal endoscopy avoided^7^ |  | Patients displaying COVID-19 symptoms should have appointments postponed at least 14 days^7^ |  |
| 8 March 2020 to 14 March 2020 | Bronchoscopy seen as high risk^8^ |  | No relevant studies identified from targeted searches |  |
| 15 March 2020 to 21 March 2020 | COVID-19 cases should undergo surgery in separating operating theatres with negative-pressure^9^; Endoscopy should be performed in negative-pressure if available^10^ | PPE required for endoscopy^10^ |  |  |
| 22 March 2020 to 31 March 2020 | Gastrointestinal endoscopy seen as high risk^11^; Precautions required for any airway management^12^; Infection control measures to be taken in operating theatre^13^ | Properly-fitted respirators should be used, then surgical masks if not available^14^; May be minimal SARS-CoV-2 contamination of PPE healthcare workers treating COVID-19 patients^15^***** | In COVID-19 positive patients, gynaecological cancer surgery should be postponed for 15 days^16^; All urology surgery should be deferred if possible^17^ |  |
| 1 April 2020 to 7 April 2020 | Endoscopy should be limited to urgent cases^18^; Precautions should accompany patient entry and exit from operating theatre^19^; Potential viral burden of surgical sites, COVID-19 risk of patient and risk of aerosolisation should be considered^20^; Procedures of the upper aerodigestive tract are most at risk^21^; Tracheostomy can be classified as high risk based on SARS outbreak.^22^; Endonasal instrumentation is aerosol risk, cold surgical instrumentation and microdebrider may reduce^23^ | Both surgical masks and cotton masks are ineffective in preventing SARS-CoV-2 transmission from patient cough^24^; Medical masks and N95 respirators have similar protection during non-AGPs, so N95s should be preserved for AGPs^25^; Staff should have donning and doffing training, and hospital supply of PPE should be regularly monitored^18^; No significant difference in instructor-led training versus video training of donning and doffing^26^; PPE associated with headaches that could compromise staff performance^27^ | Acute care surgeons should be deployed^28^; Ambulatory surgery may still have role during the pandemic^29^; Stable surgical disease should be rescheduled and have multidisciplinary decision-making^30^; Endoscopy should be deferred indefinitely, and staff and resources reallocated to prepare for surgery in healthcare demand^18^; Oncological surgery triage must account for differing rates of tumour growth^31^; Non-cancer colorectal procedures should be stopped^19^; Consider postponing spinal surgery and looking to minimise postoperative length of stay^32^ | Surgical services must develop a COVID-19 response plan that includes provision of staff training, team-based approach for emergency services, recognition and management of COVID-19 cases, and preparation for a backlog of operations^33^; Recommended PPE for orthopaedic surgeons during COVID-19 should consist of level 4 surgical gowns, face shields or goggles, double gloves, FFP2-3 or N95-99 respirators^34^; For oral and maxillofacial surgery, no elective surgery should be performed with urgent procedures postponed for recovering COVID-19 patients, specialties that overlap should work together to utilise resources efficiently, and negative pressure and PPE must be used in the operating theatre^35^ |
| 8 April 2020 to 14 April 2020 | Number of staff within operating theatre should be reduced^36^; Anaesthetic induction should occur in separate room^37^; Electrosurgical instruments should be set at the lowest possible power and not used for long continuous periods to reduce surgical smoke^38^; Theoretical risk of aerosol transmission during laparoscopy must be balanced with surgical benefits^39^; Orthopaedic surgeons may be at risk of aerosol exposure^40^ | Staff should wear surgical masks in all clinical areas^36^; Staff should identify themselves when wearing PPE^41^; Procedures for periprocedural donning and doffing published for non-surgical specialty^42^; Staff should wear full PPE for ENT surgery^43^ | Consider placing staffing in alternate roles^36^; Acute or disabling orthopaedic injuries should be operated on^44^; Foetal surgery must go ahead regardless of COVID-19 status^45^; In locations of high COVID-19 prevalence, reduce operating theatre volume and reduce staffing with backups if required^46^; Surgical services can still be affected even if no COVID-19 positive patients present^47^; Scoring systems should be considered in situations of resource scarcity^48^; Resources must be considered in triage^49^ |  |
| 15 April 2020 to 21 April 2020 | Some recommended open colorectal emergency surgery over laparosopic surgery^50^; Staff exposed to oxygen cannulas or noninvasive ventilation may be at aerosolisation risk^51^;  Oral and maxillofacial procedures may be at risk^35^ | Common breaches in donning and doffing must be identified and taught^52^; Multiple PPE modifications may lead to increased protection and staff satisfaction^53^ | Elective and endoscopic metabolic and bariatric surgery should be postponed^54^;  Reduce non-urgent activities in departments, have team-based approach for emergencies and treat COVID-19 patients as emergency patients^33^; Urgent endoscopy should continue for inflammatory bowel diseases^55^; One-third of major urological cancer surgery may require high-priority^56^; Delay of trauma surgery should be minimised^57^; For paediatric orthopaedics, developmental or chronic conditions may be delayed 2-4 months^58^ |  |
| 22 April 2020 to 30 April 2020 | Use of drills in ENT surgery can result in aerosolisation^59^; Head and neck osteotomies may result in droplet formation^60^; Use of power tools, pulsatile lavage and electrocautery in orthopaedic surgery are AGPs^34^; Risk of nosocomial COVID-19 infection for digestive surgery^61^; Recommendations for high-risk surgery must be situation-specific^62^ | Staff safety concern may vary according to PPE resource supply^63^; Standard and powered respirators do not significantly prolong simulated intubation procedures^64^ | Emergency surgery may reduce but with more severe presentations^65^; Urology services can successfully adapt to pandemic^66^; Multidisciplinary team needs to include clinical and non-clinical staff^67^; Head and neck mucosal malignancies still require surgery^68^; Delaying high-risk cancer surgery over 6 weeks can have long-term effects^69^;  Neurosurgery volume dropped more than 50%, and fast-evolving neuro-oncological operations are non-emergent presentations that have highest risk in postponement^70^; For lung cancer, delay screening, defer surveillance imaging and non-urgent surgery^71^; Considerable reduction in head and neck cancer operations^72^ |  |
| 1 May 2020 to 7 May 2020 | Aerosol can be generated in lung procedures^73^; Use hand drill or saw to decrease orthopaedic surgery aerosol^74^; Insufficient evidence to quantify COVID-19 transmission in surgical smoke but should minimise smoke through evacuators^75^; Negative pressure may reduce aerosol and droplet contamination^76^ | Face shields must be regularly sterilised^77^; Risk-adapted approach for PPE shortages^78^; Hydrogen peroxide may present scalable method of disinfecting PPE^79^; N95s are superior to surgical masks in laboratory testing, equivalent in outpatient setting, and may be superior in inpatient setting^80^; | Major cutbacks occurred in orthopaedic surgery^81, 82^; Reconstructive plastic surgery should be delayed unless urgent^83^; Many head and neck cancer operations have been cancelled^84^; Restructuring can support resident-led procedural response teams^85^; Telemedicine can be used for triaging^86^ | For head and neck and otolaryngologists, only essential surgery should be performed, and should be done so with PPE that protects against aerosol including goggles, N95s, face masks and blood-repelling gown and gloves, telemedicine is useful if resources allow^86^; access to operating theatre should be almost exclusively restricted to emergencies and oncological procedures, and use of laparoscopy in COVID-19 patients should be cautiously considered, for care of COVID-19 patients PPE with aerosol precautions must be worn^87^; ‘elective’ neurosurgical operations should be reduced, neurosurgical illnesses with risk of progression should be scheduled more promptly, and telemedicine might allow for early screening of patients^88^; any surgery that can be delayed should be postponed, surgical staff should be reduced to the essential members and provided with institutional psychological support^89^; when prioritising oncologic cases, clinicians must balance delay in cancer diagnosis or treatment against risk of COVID-19 exposure, a tailored approach to laparoscopic surgery is suggested with a case-by-case evaluation and appropriate PPE^90^; in urology, all elective surgery should be deferred in regions of high COVID-19 prevalence however operations should still be considered for urological emergencies^91^; many perioperative guidelines are contradictory and based on anecdotal data at best, surgical services need a contingency plan in an ongoing or postpandemic phase^92^; for transnasal surgery, operating theatre staff should be reduced and trainees prohibited from participating in single-surgeon operations, only COVID-19 patients will be permitted to use a COVID-19-positve operating theatre, nonemergent cases should not be cancelled indiscriminately and should be considered alongside hospital resources^93^; all recommendations for operating theatre practice are based on low levels of evidence, and although few related to personnel factors, there was consensus that the use of PPE should be mandatory^94^ |
| 8 May 2020 to 14 May 2020 | Little evidence to inform modification of operating theatre practice^94^; Decontamination and laminar airflow should occur within operating theatres^95^; Mastoidectomy with drill is an AGP but a barrier drape may reduce particle dispersion^96^; HEPA filters should be used in high-risk AGPs^97^; Colon capsule endoscopy may be considered in place of colonoscopy^98^; No clear evidence of COVID-19 in surgical plume by electrocautery or pneumoperitoneum, but should filter particles and reduce operating theatre personnel.^99^; CO_2_ laser ablation, use of high speed rotating devices, electrocautery and endotracheal suction are AGPs^100^ | Surgery should not proceed with inadequate PPE^101^; Dermatological pressure ulcers associated with extended N95 use^102^; Robotic cystectomy may need most hats and masks within urology and data analysis can be used to tailor PPE distribution^103^ | May take 45 weeks to clear backlog of operations if surgical activity is increased by 20%^104^; Early implementation of precautionary measures can be effective even in a COVID-19 centre^105^; Large number of arthroplasty operations have been cancelled resulting in significant financial losses^106^; Many patients plan to reschedule required surgery as soon as possible^107^; Reduction in trauma and degenerative spinal cases during the pandemic^108^; Long-term pathophysiological effects of SARS-CoV-2 must be considered for urology^109^; Triage should also account for age and comorbidities if hospital supplies are compromised^110^; Effect of COVID-19 on the brain can factor into triage for neurosurgery^111^ |  |
| 15 May 2020 to 21 May 2020 | Intubation and extubation are high-risk^112^; Aerosol box can potentially decrease aerosol and droplets^113^; Droplet splatter may result from otologic and endocopic endonasal procedures^114, 115^ | Seal leakage is main issue with surgical masks^116^; For ENT surgery, always wear long-sleeves, masks and gloves^117^; Caution required for giving universal PPE advice to settings of limited resources^118^; PPE use should be data-driven^119^; UVC is a potential method of decontamination of PPE^120^; N95s may provide inadequate protection during cardiopulmonary resuscitation^121^ | Non-presenters increased for endoscopies as pandemic progressed^122^; Reassessment needs to occur repeatedly as pandemic progresses^123^;  Age and comorbidities need to factor into triage^124^; Avoid cross-contamination in patient procedural flow^125^; Need to establish hierarchy for cancelled surgeries^126^ |  |
| 22 May 2020 to 31 May 2020 | Avoid the use of ultrasonic scalpel in COVID-19 patients and operations of the upper airway^127^; Laparoscopy can be considered safer overall than laparotomy^128^; Specialists may have knowledge of COVID-19 less understanding regarding preventative measures^129^; Precautions are necessary for endoscopic skull base surgery^93^;  Transnasal drill and cautery may create aerosol^130^ | Surgical mask may reduce COVID-19 transmission, based on animal model^131^; Consistency within a nation’s guidelines may improve overall PPE utilisation^132^; Many healthcare workers have poor knowledge of face masks and PPE^133^; PPE and respirators are required in ophthalmic surgery^134^ | Hospital management via hub and spoke model may be ineffective for cancer surgery in high prevalence settings^135^; Lower prevalence settings may adjust easier to the effects of the pandemic^136^; Differences of public and private surgical systems must be considered^137^; There is lack of consensus amongst triaging for gastroenterologists^138^; Surgical triage should adjust for increased ICU demand and decreased volume^139^; Delaying benign tumour surgery can have long term effects on the surgical systems^140^; Some vascular surgeons were redeployed outside specialty^141^; Need to redistribute resources from non-essential settings^90^; Urgent surgery may become more challenging due to diagnostic delay^142^ |  |
| 1 June 2020 to 7 June 2020 | Prefer disposable instrumentation and cables, use low CO_2_ insufflation pressures and low power electrocautery, prefer closed-system CO_2_ insufflation and desufflation systems and avoid leaks through ports^143^; Tumour ablation procedures also require precautions^144^ | Physical distancing of at least 1 m, face mask and eye protection use could all result in reduced infection risk, with stronger associations with N95 or similar respirators compared with disposable surgical masks^145^; There may be inconsistency in intraoperative use of PPE for even high-risk operations^146^; In high prevalence settings, many staff may have inadequate PPE supply^147^; Important that staff in low-risk COVID-19 situations do not wear full PPE to allow for resource redistribution^148^; Over one-third of staff may use face masks incorrectly^149^; Cloth masks are inferior to medical masks^150^; Neurosurgical drilling and endoscopy should be done with full PPE^151^ | Considerable restriction of outpatient clinics and non-emergency surgery.^152^; Heterogeneity amongst worldwide esophagogastric cancer treatment^153^; disproportionate case reduction in spinal surgery after ban on elective surgery^154^; Pandemic can make conditions that are influenced by weight worse^155^ | For oculofacial, plastic and orbital surgery, procedures involving cautery, powered surgical instrument, laser or bone manipulation, procedures of the nose, mouth or sinuses should all be treated as high risk and use of N95 or equivalent is strongly recommended with a surgical mask, face shield and/or goggles required, procedures of the nasolacrimal system vary in their risk and PPE requirements^156^; there was consensus amongst urology guidelines on classification of some oncology cases^157^; otolaryngology patients in stable condition should be consulted using telemedicine options, with only emergency consultations and procedures being performed, mucosa-involving otolaryngologic procedures are high risk and require enhanced PPE, urgent surgery should be performed in a negative pressure operating theatre with HEPA filtration^158^; for otolaryngologic surgery, procedures and PPE requirements should be categorised according to aerosolisation risk and possibility of COVID-19 diagnosis^159^; SARS-CoV-2 has never been found in surgical smoke and there is currently no data to support its virulence in surgical smoke or aerosol, if proper evacuation of smoke measures are taken then laparoscopy is safer for the operating team than laparotomy^128^; for performing minimally invasive surgery on COVID-19 paediatric patients, consider conservative treatment where possible, dedicate a theatre, reusable laparoscopic instrumentation, prefer disposable instrumentation, use low CO_2_ insufflation pressures and low power cautery, prefer closed-systems for CO_2_ insufflation and desufflation, and avoid leaks through ports^143^; facial plastic and reconstructive surgeons are at risk and protocols must be in place to mitigate exposure risk^160^; a conservative approach is favoured for spinal conditions although no patient should be deprived of standard care owing to concerns about COVID-19, triaging should be based on underlying spinal pathology^161^; for orthopaedic surgery, strict protection at every step of the patient pathway is important to reduce risk of cross-infection^162^ |
| 8 June 2020 to 14 June 2020 | Aerosol boxes may increase intubation times, increase risk of hypoxia, and damage PPE^163^; Laparoscopic cholecystectomy remains treatment of choice for acute cholecystitis^164^; Aerosolisation may not be predominant mode of transmission for SARS-CoV-2 in hospitals, so hand hygiene and other decreases to fomite and droplet transmission are important^165^; Clear plastic drapes may limit droplet spray.^166^; Theoretical risk of virus in smoke cannot be excluded^167^ | Proper PPE usage can result in adequate protection from COVID-19, even in high-risk or high prevalence settings^168^; Staff should wear respirators continuously on shifts^169^; Proper PPE can reduce infection, but infection may be mainly related to extrahospital contact^170^; Staff communication and performance may be affected by PPE^171^ | Telemedicine can be used to reduce outpatient and followup volume^172^ |  |
| 15 June 2020 to 21 June 2020 | Some oculofacial procedures are AGPs^156^; Conflicting stances on use of energy devices and laparoscopy exist, and risk of SARS-CoV-2 aerosol transmission is biologically plausible^173^ | Previous coronavirus experiences can be drawn upon for cloth, N95 and surgical masks guidance^174^ | Different measures should be ready for implementation at different cumulative incidences of COVID-19^175^; Paediatric departments may have to merge with adult departments during COVID-19^176^; Heterogeneity in viewpoints amongst hospitals for scheduling PPE and also testing for COVID-19^177^; Bariatric and metabolic surgery triage should factor in obesity and type 2 diabetes mellitus as these are COVID-19 risk factors^178^ |  |
| 22 June 2020 to 30 June 2020 | Use of smaller tip during corneal phacoemulsification surgery can reduce aerosol generation^179^; Vitrectomy may not result in aerosol generation^180^; May be little risk of intraoperative SARS-CoV-2 exposure from abdominal viscera or fluid^181^ | N95s are superior in reducing SARS transmission compared to surgical masks in healthcare settings, however it is uncertain whether this translates to COVID-19^182^; Paucity of strong evidence that increased surgical mask can increase fomite transmission^183^; Multilayered cloth masks may offer increased aerosol protection^184^ | Decrease in overall patients but increase in admissions ratio^185^; For heart and vascular surgery, a hub and spoke organisational model may be effective in high prevalence settings^186^; For urology, the greatest amount of reorganisation occurred in Europe^187^;  In urology there may be considerable increases in oncological and urgent presentations^188^ |  |
| 1 July 2020 to 7 July 2020 | Appropriate suction irrigator system and HEPA filters should be used during any procedure that either generates surgical smoke or creates aerosol^189^;  Even in high prevalence settings, intraoperative COVID-19 transmission can be minimised with adequate preoperative screening^190^; Local irrigation can reduce particle generation in craniomaxillofacial trauma surgery^191^ | The use of heat and humidity may be a scalable method of decontaminating N95 respirators^192^; Autoclave sterilisation and ethanol treatment of masks and N95s may compromise performance and integrity^193^; Simulation can be used as a tool for learning and assessing PPE use^194^; Difference in protective measures between university and non-university hospitals must be considered^195^ | Countries with high COVID-19 prevalence reported inadequate critical care capacity^196^; Majority of operations may be cancelled by patient renunciation if in setting of high prevalence^197^ | Systematic review not conducted |
| 8 July 2020 to 14 July 2020 | In high prevalence setting, 1% of endoscopic surgeons were infected by COVID-19^198^; Operating theatre staff should only enter after anaesthetic induction and particulates are all cleared.^199^ | Within Australia and New Zealand hospitals, there was a lack of reinforcement of PPE training^200^; PPE is required for vitreoretinal surgery^201^; Head and dorsum of the foot may be areas of PPE most likely to be contaminated^202^; Availability of PPE may result in lower psychological stress for staff^203^; Reduced number of staff operating theatre may also decrease overall PPE usage^204^; No comparative trials of different masks against COVID-19 have been conducted^205^; Extended use of PPE can reduce overall PPE consumption^206^; Full PPE may not impede colonoscopy performance^207^ | Data-driven assessment of resources allows for rapid redistribution^208^; Interhospital differences in practice and resources prior to COVID-19 may contribute to inefficiency in COVID-19 adaptation^209^ |  |
| 15 July 2020 to 21 July 2020 | Shield required for slit-lamp exam in ophthalmic procedures^210^; Potential AGP timepoints during operations can be identified to improve safety^211^ | Suspected or confirmed COVID-19 patients should wear mask during transfer to operating theatre^212^; Putting surgical mask over N95 may prolong use^213^; Homemade masks may provide a backup to surgical masks if low resource supply^214^; If exposed to the COVID-19 patients with insufficient PPE, staff should quarantine^215^; Decontaminating N95 respirators with ultraviolet germicidal irradiation may not decrease efficacy or safety^216^ | Time periods for stages of triage should be identified and also be patient-focused^217^; Carefully staged return to elective surgery should align with local COVID-19 caseload^218^; Resources should be projected prior to reinstating elective orthopaedic surgery^219^; Close cooperation with the hospital administrative staff may improve surgical triage^220, 221^; Oncologically urgent colorectal cancer cases may be delayed 6-12 weeks without jeopardising patient outcomes^222^ |  |
| 22 July 2020 to 31 July 2020 | HEPA filters differ in function^223^; Multiple commercially available surgical smoke evacuators, can be used to minimise surgical plume^224^; Decrease in operating theatre time during the pandemic^225^; Conjunctival and lacrimal sac operations require N95 respirators^226^; Operating theatre should be cleaned postoperatively and adequate time allowed for air to be cycled^227^; Avoid diathermy, pulse lavage and ultrasonic tools to minimise aerosolisation^228^; Insufficient evidence exists to recommend either open or laparoscopic approach^229^ | Extensive testing of staff, with proactive tracing and quarantining can minimise spread within departments^230^; Household materials, including vacuum cleaner bags, may be alternatives to surgical masks during situations of low supply^231^; N95 reprocessing may be best using moist heat or vaporous hydrogen peroxide^232^; Low-level evidence supports the use of ultraviolet germicidal irradiation, moist heat, microwave-generated steam, and hydrogen peroxide for decontamination and reuse of N95s^233^; Tele-rounding is possible if necessary^234^; Perceived comforts of PPE resource supply levels is associated with the immediate availability of N95s^235^; PAPRs led to hearing impairment that is comparable to moderate-severe hearing loss^236^ | Delaying paediatric surgery may affect neurocognitive development^237^; Swift implementation of triage protocols may create better postoperative outcomes^238^; Progressive integration of inactive specialists can relieve front-line colleagues’ burden^239^; Postoperative length of stay can be reduced with a multidisciplinary approach^240^; Dedicated COVID-19 centres in high prevalence locations are severely affected^241^ |  |
| 1 August 2020 to 7 August 2020 | For ENT surgeons with COVID-19, source of infection may stem equally from clinical activity and community spread^242^; There is a significant risk of acquiring COVID-19 from performing intubation^243^ | Stockpiled N95 and surgical masks may be beyond expiry date and prone to protection flaws^244^;  PPE is essential for psychological wellbeing of staff^245^; Non-compliance with PPE is mostly seen for eye protection^246^; N95s create higher facial skin temperature and discomfort and lower wearing adherence compared to surgical masks^247^ | Local resources can be redistributed for surgery^248^; COVID-free hospitals can allow elective surgery to safely continue even amidst high COVID-19 prevalence^249^; Hospital stay patients should receive clear instructions of modified protocols^250^; Most head and neck oncology surgeons did not modify practice significantly^251^ |  |

*According to date of database entry

**Synthesis of data from rapid review

***Synthesis of data from systematic review

****Date of identification of SARS-CoV-2 by World Health Organization^252^

*****First study, chronologically, that ranked above ‘poor’ for all dimensions within Evans’ hierarchy of evidence^4^

**Table S4. Evaluation of initial evidence-based recommendations (April 2020)^189, 212, 218, 253-255^**

| **Recommendation as of April 2020** | **Recommendation as of August 2020** |
| --- | --- |
| **Safe intraoperative practice^189, 253^** | |
| With respect to testing for COVID-19 and PPE use, the recommendation is that local protocols for risk stratification should be followed. | **No strong evidence** to justify significant change in recommendation |
| There is no current evidence that laparoscopy presents a greater risk to the surgical team in the operating theatre than open surgery with respect to viruses, but it is important to maintain a level of caution due to the possibility of aerosolisation | **No strong evidence** to justify significant change in recommendation |
| During all procedures a reduction in occupational exposure to surgical plume is advisable using an appropriate capture device. There is evidence that all energy sources which produce a surgical plume during surgery may facilitate viral transmission. Limited use of lower energy devices may reduce the viral load and should be the preferred option. | **No strong evidence** to justify significant change in recommendation |
| Specifically for laparoscopic surgery, desufflation of pneumoperitoneum must be performed via an appropriate suction device attached to a HEPA filter to prevent venting into the operating theatre, for example an insufflation-filtration device. Otherwise other methods need to be employed to reduce any potential release. | **No strong evidence** to justify significant change in recommendation |
| SARS-CoV-2 has been observed in faecal cultures; viral component staining and replication products have been detected in gastrointestinal epithelium; RT-PCR has detected the SARS-CoV-2 RNA genome in peritoneal fluid; there is equivocal evidence of viral presence in blood, while early studies so far have not found evidence of presence in urine. However, all tissues and bodily fluids should be treated as a potential virus source. | **No strong evidence** to justify significant change in recommendation |
| **Personal protective equipment^212, 254^** | |
| Implement mandatory infectious disease control training for all surgical staff | **No strong evidence** to justify significant change in recommendation |
| Implement mandatory PPE donning and doffing training for all surgical staff | **No strong evidence** to justify significant change in recommendation |
| Develop contingency plans to extend the use of PPE, especially P2/N95 respirators | **No strong evidence** to justify significant change in recommendation |
| Where possible, patients with severe respiratory symptoms and/or suspected or confirmed COVID-19 to wear surgical masks during transfer | **No strong evidence** to justify significant change in recommendation |
| PPE for procedures that are not aerosol-generating: surgical mask, disposable gown, disposable double sterile gloves, eye protection (safety glasses, goggles or full-face shield), head covering, shoe covering, proper hand hygiene | **No strong evidence** to justify significant change in recommendation |
| PPE for AGPs: surgical P2/N95 respirator, disposable fluid-impervious long-sleeved gown, disposable fluid-impervious apron, disposable double sterile gloves, eye protection (safety glasses, goggles or full-face shield), Bouffant head covering, disposable impervious shoe covering, proper hand hygiene | **No strong evidence** to justify significant change in recommendation |
| PPE for emergency surgery (within 24 hours of presentation): treat patient as COVID-19 positive until diagnostic tests indicate otherwise, surgical staff to don the same PPE as worn for AGPs | **No strong evidence** to justify significant change in recommendation |
| PPE for category 1 elective surgery (within 1 month of presentation): if patient is COVID-19 positive, surgical staff to don the same PPE as worn for AGPs, if patient confirmed COVID-19 negative, surgical staff to don PPE as outlined by the surgical unit of their individual healthcare facility | **No strong evidence** to justify significant change in recommendation |
| **Surgical triage^218, 255^** | |
| Urgent and emergency surgery must continue, with appropriate precautions | **No strong evidence** to justify significant change in recommendation |
| Limit non-urgent elective surgery only to cases where delay would significantly worsen patient outcome; before scheduling, particularly for complex procedures, obtain a documented, independent peer or Surgical Review Committee opinion where possible | **No strong evidence** to justify significant change in recommendation, however only applicable to settings of high COVID-19 prevalence |
| Multidisciplinary care, decision-making and review should be implemented wherever possible, especially for complex surgical issues; where multidisciplinary team discussion is not possible a discussion with a colleague should be documented | **No strong evidence** to justify significant change in recommendation |
| Surgical decision-making should incorporate a real-time, data-driven approach of daily assessment that considers both individual patient cases and the supply of hospital resources, particularly PPE | **No strong evidence** to justify significant change in recommendation |
| Case-by-case assessment of immediate need to operate should incorporate the individual patient’s age, comorbidities, disease severity, wishes, and possibility of COVID-19 infection | **No strong evidence** to justify significant change in recommendation |
| Nonoperative management strategies that do not worsen patient outcomes should be considered where possible, based on the surgical team’s decision | **No strong evidence** to justify significant change in recommendation, however only applicable to settings of high COVID-19 prevalence |
| Minimise postoperative length of stay and need for critical care, encouraging use of enhanced recovery protocols and maximising use of short stay procedures where possible | **No strong evidence** to justify significant change in recommendation, however only applicable to settings of high COVID-19 prevalence |
| Senior consultants should direct both the initial assessment of surgical patients and an efficient and appropriate management strategy, oversighting the operative intervention | **No strong evidence** to justify significant change in recommendation, however only applicable to settings of high COVID-19 prevalence |
| Preparation for a potential future surge in COVID-19 cases is crucial | **No strong evidence** to justify significant change in recommendation |
| Avoid unnecessary delay of cancer cases, as a backlog could progress to a future peak of urgent cases that may coincide with a peak in COVID-19 cases | **No strong evidence** to justify significant change in recommendation |
| Patients should be assessed on booking and then again on admission for COVID-19 status; this would include questions related to a history of symptoms, close contacts and recent travel. COVID-19 testing should be carried out wherever possible in accordance with current Federal or State guidelines | **No strong evidence** to justify significant change in recommendation |

**References**

1. Carracedo D, Alcaraz A, Allona A, et al. [Robotic and laparosocpic urological surgery during COVID-19 pandemia.]. *Arch Esp Urol*. Jun 2020;73(5):463-470. Situacion de la cirugia laparoscopica y robotica urologica durante la pandemia COVID-19.

2. Puche-Sanz I, Sabio-Bonilla A, Sanchez-Conde V, et al. [Emergency surgery during COVID-19 pandemia.]. *Arch Esp Urol*. Jun 2020;73(5):353-359. Cirugia de urgencia en urologia durante la pandemia Covid-19.

3. Dalkey N, Helmer O. An experimental application of the Delphi method to the use of experts. *Management Science*. 1963;9(3):458-467.

4. Evans D. Hierarchy of evidence: a framework for ranking evidence evaluating healthcare interventions. *J Clin Nurs*. Jan 2003;12(1):77-84. doi:10.1046/j.1365-2702.2003.00662.x

5. Yu GY, Lou Z, Zhang W. [Several suggestion of operation for colorectal cancer under the outbreak of Corona Virus Disease 19 in China]. *Zhonghua Wei Chang Wai Ke Za Zhi*. Feb 19 2020;23(3):9-11. doi:10.3760/cma.j.issn.1671-0274.2020.03.002

6. Chen YH, Peng JS. [Treatment strategy for gastrointestinal tumor under the outbreak of novel coronavirus pneumonia in China]. *Zhonghua Wei Chang Wai Ke Za Zhi*. Feb 25 2020;23(2):I-IV. doi:10.3760/cma.j.issn.1671-0274.2020.02.001

7. Lai THT, Tang EWH, Chau SKY, Fung KSC, Li KKW. Stepping up infection control measures in ophthalmology during the novel coronavirus outbreak: an experience from Hong Kong. *Graefes Arch Clin Exp Ophthalmol*. May 2020;258(5):1049-1055. doi:10.1007/s00417-020-04641-8

8. Group of Interventional Respiratory Medicine Chinese Thoracic Society. [Expert consensus for bronchoscopy during the epidemic of 2019 novel coronavirus infection (Trial version)]. *Zhonghua Jie He He Hu Xi Za Zhi*. Mar 12 2020;43(3):199-202. doi:10.3760/cma.j.issn.1001-0939.2020.03.012

9. Luo Y, Zhong M. [Standardized diagnosis and treatment of colorectal cancer during the outbreak of corona virus disease 2019 in Renji hospital]. *Zhonghua Wei Chang Wai Ke Za Zhi*. Mar 25 2020;23(3):211-216. doi:10.3760/cma.j.cn.441530-20200217-00057

10. Repici A, Maselli R, Colombo M, et al. Coronavirus (COVID-19) outbreak: what the department of endoscopy should know. *Gastrointest Endosc*. Jul 2020;92(1):192-197. doi:10.1016/j.gie.2020.03.019

11. Sociedad Espanola de Patologia Digestiva, Asociacion Espanola de Gastroenterologia. Recommendations by the SEPD and AEG, both in general and on the operation of gastrointestinal endoscopy and gastroenterology units, concerning the current SARS-CoV-2 pandemic (March, 18). *Rev Esp Enferm Dig*. Apr 2020;112(4):319-322. doi:10.17235/reed.2020.7052/2020

12. Cook TM, El-Boghdadly K, McGuire B, McNarry AF, Patel A, Higgs A. Consensus guidelines for managing the airway in patients with COVID-19: Guidelines from the Difficult Airway Society, the Association of Anaesthetists the Intensive Care Society, the Faculty of Intensive Care Medicine and the Royal College of Anaesthetists. *Anaesthesia*. Jun 2020;75(6):785-799. doi:10.1111/anae.15054

13. Dexter F, Parra MC, Brown JR, Loftus RW. Perioperative COVID-19 Defense: An Evidence-Based Approach for Optimization of Infection Control and Operating Room Management. *Anesth Analg*. Jul 2020;131(1):37-42. doi:10.1213/ANE.0000000000004829

14. Chughtai AA, Seale H, Islam MS, Owais M, Macintyre CR. Policies on the use of respiratory protection for hospital health workers to protect from coronavirus disease (COVID-19). *Int J Nurs Stud*. May 2020;105:103567. doi:10.1016/j.ijnurstu.2020.103567

15. Ong SWX, Tan YK, Sutjipto S, et al. Absence of contamination of personal protective equipment (PPE) by severe acute respiratory syndrome coronavirus 2 (SARS-CoV-2). *Infect Control Hosp Epidemiol*. May 2020;41(5):614-616. doi:10.1017/ice.2020.91

16. Akladios C, Azais H, Ballester M, et al. [Guidelines for surgical management of gynaecological cancer during pandemic COVID-19 period - FRANCOGYN group for the CNGOF]. *Gynecol Obstet Fertil Senol*. May 2020;48(5):444-447. Prise en charge chirurgicale des cancers gynecologiques en periode de pandemie COVID-19 - Recommandations du Groupe FRANCOGYN pour le CNGOF. doi:10.1016/j.gofs.2020.03.017

17. Ficarra V, Novara G, Abrate A, et al. Urology practice during the COVID-19 pandemic. *Minerva Urol Nefrol*. Jun 2020;72(3):369-375. doi:10.23736/S0393-2249.20.03846-1

18. Chiu PWY, Ng SC, Inoue H, et al. Practice of endoscopy during COVID-19 pandemic: position statements of the Asian Pacific Society for Digestive Endoscopy (APSDE-COVID statements). *Gut*. Jun 2020;69(6):991-996. doi:10.1136/gutjnl-2020-321185

19. Di Saverio S, Pata F, Gallo G, et al. Coronavirus pandemic and colorectal surgery: practical advice based on the Italian experience. *Colorectal Dis*. Jun 2020;22(6):625-634. doi:10.1111/codi.15056

20. Forrester JD, Nassar AK, Maggio PM, Hawn MT. Precautions for Operating Room Team Members During the COVID-19 Pandemic. *J Am Coll Surg*. Jun 2020;230(6):1098-1101. doi:10.1016/j.jamcollsurg.2020.03.030

21. Givi B, Schiff BA, Chinn SB, et al. Safety Recommendations for Evaluation and Surgery of the Head and Neck During the COVID-19 Pandemic. *JAMA Otolaryngol Head Neck Surg*. Mar 31 2020;doi:10.1001/jamaoto.2020.0780

22. Tay JK, Khoo ML, Loh WS. Surgical Considerations for Tracheostomy During the COVID-19 Pandemic: Lessons Learned From the Severe Acute Respiratory Syndrome Outbreak. *JAMA Otolaryngol Head Neck Surg*. Mar 31 2020;doi:10.1001/jamaoto.2020.0764

23. Workman AD, Welling DB, Carter BS, et al. Endonasal instrumentation and aerosolization risk in the era of COVID-19: simulation, literature review, and proposed mitigation strategies. *Int Forum Allergy Rhinol*. Jul 2020;10(7):798-805. doi:10.1002/alr.22577

24. Bae S, Kim MC, Kim JY, et al. Effectiveness of Surgical and Cotton Masks in Blocking SARS-CoV-2: A Controlled Comparison in 4 Patients. *Ann Intern Med*. Jul 7 2020;173(1):W22-W23. doi:10.7326/M20-1342

25. Bartoszko JJ, Farooqi MAM, Alhazzani W, Loeb M. Medical masks vs N95 respirators for preventing COVID-19 in healthcare workers: A systematic review and meta-analysis of randomized trials. *Influenza Other Respir Viruses*. Jul 2020;14(4):365-373. doi:10.1111/irv.12745

26. Christensen L, Rasmussen CS, Benfield T, Franc JM. A Randomized Trial of Instructor-Led Training Versus Video Lesson in Training Health Care Providers in Proper Donning and Doffing of Personal Protective Equipment. *Disaster Med Public Health Prep*. Mar 30 2020:1-15. doi:10.1017/dmp.2020.56

27. Ong JJY, Bharatendu C, Goh Y, et al. Headaches Associated With Personal Protective Equipment - A Cross-Sectional Study Among Frontline Healthcare Workers During COVID-19. *Headache*. May 2020;60(5):864-877. doi:10.1111/head.13811

28. Ross SW, Lauer CW, Miles WS, et al. Maximizing the Calm before the Storm: Tiered Surgical Response Plan for Novel Coronavirus (COVID-19). *J Am Coll Surg*. Jun 2020;230(6):1080-1091 e3. doi:10.1016/j.jamcollsurg.2020.03.019

29. Rajan N, Joshi GP. COVID-19: Role of Ambulatory Surgery Facilities in This Global Pandemic. *Anesth Analg*. Jul 2020;131(1):31-36. doi:10.1213/ANE.0000000000004847

30. Besnier E, Tuech JJ, Schwarz L. We Asked the Experts: Covid-19 Outbreak: Is There Still a Place for Scheduled Surgery? "Reflection from Pathophysiological Data". *World J Surg*. Jun 2020;44(6):1695-1698. doi:10.1007/s00268-020-05501-6

31. Tuech JJ, Gangloff A, Di Fiore F, et al. Strategy for the practice of digestive and oncological surgery during the Covid-19 epidemic. *J Visc Surg*. Jun 2020;157(3S1):S7-S12. doi:10.1016/j.jviscsurg.2020.03.008

32. Donnally CJ, 3rd, Shenoy K, Vaccaro AR, Schroeder GD, Kepler CK. Triaging Spine Surgery in the COVID-19 Era. *Clin Spine Surg*. May 2020;33(4):129-130. doi:10.1097/BSD.0000000000000988

33. COVIDSurg Collaborative. Global guidance for surgical care during the COVID-19 pandemic. *Br J Surg*. Apr 15 2020;doi:10.1002/bjs.11646

34. Hirschmann MT, Hart A, Henckel J, Sadoghi P, Seil R, Mouton C. COVID-19 coronavirus: recommended personal protective equipment for the orthopaedic and trauma surgeon. *Knee Surg Sports Traumatol Arthrosc*. Jun 2020;28(6):1690-1698. doi:10.1007/s00167-020-06022-4

35. Zimmermann M, Nkenke E. Approaches to the management of patients in oral and maxillofacial surgery during COVID-19 pandemic. *J Craniomaxillofac Surg*. May 2020;48(5):521-526. doi:10.1016/j.jcms.2020.03.011

36. Ahmed S, Tan WLG, Chong YL. Surgical Response to COVID-19 Pandemic: A Singapore Perspective. *J Am Coll Surg*. Jun 2020;230(6):1074-1077. doi:10.1016/j.jamcollsurg.2020.04.003

37. Awad ME, Rumley JCL, Vazquez JA, Devine JG. Perioperative Considerations in Urgent Surgical Care of Suspected and Confirmed COVID-19 Orthopaedic Patients: Operating Room Protocols and Recommendations in the Current COVID-19 Pandemic. *J Am Acad Orthop Surg*. Jun 1 2020;28(11):451-463. doi:10.5435/JAAOS-D-20-00227

38. Ciavattini A, Delli Carpini G, Giannella L, et al. Expert consensus from the Italian Society for Colposcopy and Cervico-Vaginal Pathology (SICPCV) for colposcopy and outpatient surgery of the lower genital tract during the COVID-19 pandemic. *Int J Gynaecol Obstet*. Jun 2020;149(3):269-272. doi:10.1002/ijgo.13158

39. Di Saverio S, Khan M, Pata F, et al. Laparoscopy at all costs? Not now during COVID-19 outbreak and not for acute care surgery and emergency colorectal surgery: A practical algorithm from a hub tertiary teaching hospital in Northern Lombardy, Italy. *J Trauma Acute Care Surg*. Jun 2020;88(6):715-718. doi:10.1097/TA.0000000000002727

40. Guo X, Wang J, Hu D, et al. Survey of COVID-19 Disease Among Orthopaedic Surgeons in Wuhan, People's Republic of China. *J Bone Joint Surg Am*. May 20 2020;102(10):847-854. doi:10.2106/JBJS.20.00417

41. Balibrea JM, Badia JM, Rubio Perez I, et al. Surgical Management of Patients With COVID-19 Infection. Recommendations of the Spanish Association of Surgeons. *Cir Esp*. May 2020;98(5):251-259. Manejo quirurgico de pacientes con infeccion por COVID-19. Recomendaciones de la Asociacion Espanola de Cirujanos. doi:10.1016/j.ciresp.2020.03.001

42. John TJ, Hassan K, Weich H. Donning and doffing of personal protective equipment (PPE) for angiography during the COVID-19 crisis. *Eur Heart J*. May 14 2020;41(19):1786-1787. doi:10.1093/eurheartj/ehaa283

43. Kowalski LP, Sanabria A, Ridge JA, et al. COVID-19 pandemic: Effects and evidence-based recommendations for otolaryngology and head and neck surgery practice. *Head Neck*. Jun 2020;42(6):1259-1267. doi:10.1002/hed.26164

44. DePhillipo NN, Larson CM, O'Neill OR, LaPrade RF. Guidelines for Ambulatory Surgery Centers for the Care of Surgically Necessary/Time-Sensitive Orthopaedic Cases During the COVID-19 Pandemic. *J Bone Joint Surg Am*. Jun 3 2020;102(11):933-936. doi:10.2106/JBJS.20.00489

45. Deprest J, Van Ranst M, Lannoo L, et al. SARS-CoV2 (COVID-19) infection: is fetal surgery in times of national disasters reasonable? *Prenat Diagn*. Apr 11 2020;doi:10.1002/pd.5702

46. Lancaster EM, Sosa JA, Sammann A, et al. Rapid Response of an Academic Surgical Department to the COVID-19 Pandemic: Implications for Patients, Surgeons, and the Community. *J Am Coll Surg*. Jun 2020;230(6):1064-1073. doi:10.1016/j.jamcollsurg.2020.04.007

47. McBride KE, Brown KGM, Fisher OM, Steffens D, Yeo DA, Koh CE. Impact of the COVID-19 pandemic on surgical services: early experiences at a nominated COVID-19 centre. *ANZ J Surg*. May 2020;90(5):663-665. doi:10.1111/ans.15900

48. Prachand VN, Milner R, Angelos P, et al. Medically Necessary, Time-Sensitive Procedures: Scoring System to Ethically and Efficiently Manage Resource Scarcity and Provider Risk During the COVID-19 Pandemic. *J Am Coll Surg*. Aug 2020;231(2):281-288. doi:10.1016/j.jamcollsurg.2020.04.011

49. Stensland KD, Morgan TM, Moinzadeh A, et al. Considerations in the Triage of Urologic Surgeries During the COVID-19 Pandemic. *Eur Urol*. Jun 2020;77(6):663-666. doi:10.1016/j.eururo.2020.03.027

50. Angelos G, Dockter AG, Gachabayov M, Latifi R, Bergamaschi R. Emergency Colorectal Surgery in a COVID-19 Pandemic Epicenter. *Surg Technol Int*. May 28 2020;36:18-21.

51. Ferioli M, Cisternino C, Leo V, Pisani L, Palange P, Nava S. Protecting healthcare workers from SARS-CoV-2 infection: practical indications. *Eur Respir Rev*. Mar 31 2020;29(155)doi:10.1183/16000617.0068-2020

52. Munoz-Leyva F, Niazi AU. Common breaches in biosafety during donning and doffing of protective personal equipment used in the care of COVID-19 patients. *Can J Anaesth*. Jul 2020;67(7):900-901. doi:10.1007/s12630-020-01648-x

53. Verbeek JH, Rajamaki B, Ijaz S, et al. Personal protective equipment for preventing highly infectious diseases due to exposure to contaminated body fluids in healthcare staff. *Cochrane Database Syst Rev*. Apr 15 2020;4:CD011621. doi:10.1002/14651858.CD011621.pub4

54. Yang W, Wang C, Shikora S, Kow L. Recommendations for Metabolic and Bariatric Surgery During the COVID-19 Pandemic from IFSO. *Obes Surg*. Jun 2020;30(6):2071-2073. doi:10.1007/s11695-020-04578-1

55. Iacucci M, Cannatelli R, Labarile N, et al. Endoscopy in inflammatory bowel diseases during the COVID-19 pandemic and post-pandemic period. *Lancet Gastroenterol Hepatol*. Jun 2020;5(6):598-606. doi:10.1016/S2468-1253(20)30119-9

56. Campi R, Amparore D, Capitanio U, et al. Assessing the Burden of Nondeferrable Major Uro-oncologic Surgery to Guide Prioritisation Strategies During the COVID-19 Pandemic: Insights from Three Italian High-volume Referral Centres. *Eur Urol*. Jul 2020;78(1):11-15. doi:10.1016/j.eururo.2020.03.054

57. Coimbra R, Edwards S, Kurihara H, et al. European Society of Trauma and Emergency Surgery (ESTES) recommendations for trauma and emergency surgery preparation during times of COVID-19 infection. *Eur J Trauma Emerg Surg*. Jun 2020;46(3):505-510. doi:10.1007/s00068-020-01364-7

58. Farrell S, Schaeffer EK, Mulpuri K. Recommendations for the Care of Pediatric Orthopaedic Patients During the COVID-19 Pandemic. *J Am Acad Orthop Surg*. Jun 1 2020;28(11):e477-e486. doi:10.5435/JAAOS-D-20-00391

59. Castelnuovo P, Turri-Zanoni M, Karligkiotis A, et al. Skull-base surgery during the COVID-19 pandemic: the Italian Skull Base Society recommendations. *Int Forum Allergy Rhinol*. Aug 2020;10(8):963-967. doi:10.1002/alr.22596

60. Chow VLY, Chan JYW, Ho VWY, et al. Conservation of personal protective equipment for head and neck cancer surgery during COVID-19 pandemic. *Head Neck*. Jun 2020;42(6):1187-1193. doi:10.1002/hed.26215

61. Luong-Nguyen M, Hermand H, Abdalla S, et al. [Nosocomial infection with SARS-Cov-2 within Departments of Digestive Surgery]. *J Chir Visc*. Apr 24 2020;Infection nosocomiale a SARS-Cov-2 dans les services de Chirurgie Digestive. doi:10.1016/j.jchirv.2020.04.012

62. Panuganti BA, Pang J, Califano J, Chan JYK. Procedural precautions and personal protective equipment during head and neck instrumentation in the COVID-19 era. *Head Neck*. Jul 2020;42(7):1645-1651. doi:10.1002/hed.26220

63. Delgado D, Wyss Quintana F, Perez G, et al. Personal Safety during the COVID-19 Pandemic: Realities and Perspectives of Healthcare Workers in Latin America. *Int J Environ Res Public Health*. Apr 18 2020;17(8)doi:10.3390/ijerph17082798

64. Schumacher J, Arlidge J, Dudley D, Sicinski M, Ahmad I. The impact of respiratory protective equipment on difficult airway management: a randomised, crossover, simulation study. *Anaesthesia*. Apr 26 2020;doi:10.1111/anae.15102

65. Alvarez Gallego M, Gortazar de Las Casas S, Pascual Miguelanez I, et al. SARS-CoV-2 pandemic on the activity and professionals of a General Surgery and Digestive Surgery Service in a tertiary hospital. *Cir Esp*. Jun - Jul 2020;98(6):320-327. Impacto de la pandemia por SARS-CoV-2 sobre la actividad y profesionales de un Servicio de Cirugia General y del Aparato Digestivo en un hospital terciario. doi:10.1016/j.ciresp.2020.04.001

66. Borchert A, Baumgarten L, Dalela D, et al. Managing Urology Consultations During COVID-19 Pandemic: Application of a Structured Care Pathway. *Urology*. Jul 2020;141:7-11. doi:10.1016/j.urology.2020.04.059

67. Brethauer SA, Poulose BK, Needleman BJ, et al. Redesigning a Department of Surgery during the COVID-19 Pandemic. *J Gastrointest Surg*. Aug 2020;24(8):1852-1859. doi:10.1007/s11605-020-04608-4

68. Crosby DL, Sharma A. Evidence-Based Guidelines for Management of Head and Neck Mucosal Malignancies during the COVID-19 Pandemic. *Otolaryngol Head Neck Surg*. Jul 2020;163(1):16-24. doi:10.1177/0194599820923623

69. Finley C, Prashad A, Camuso N, et al. Guidance for management of cancer surgery during the COVID-19 pandemic. *Can J Surg*. May 1 2020;63(22):S2-S4. doi:10.1503/cjs.005620

70. Jean WC, Ironside NT, Sack KD, Felbaum DR, Syed HR. The impact of COVID-19 on neurosurgeons and the strategy for triaging non-emergent operations: a global neurosurgery study. *Acta Neurochir (Wien)*. Jun 2020;162(6):1229-1240. doi:10.1007/s00701-020-04342-5

71. Mazzone PJ, Gould MK, Arenberg DA, et al. Management of Lung Nodules and Lung Cancer Screening During the COVID-19 Pandemic: CHEST Expert Panel Report. *Chest*. Jul 2020;158(1):406-415. doi:10.1016/j.chest.2020.04.020

72. Patel RJ, Kejner A, McMullen C. Early institutional head and neck oncologic and microvascular surgery practice patterns across the United States during the SARS-CoV-2 (COVID19) pandemic. *Head Neck*. Jun 2020;42(6):1168-1172. doi:10.1002/hed.26189

73. Thornton M, Reid D, Shelley B, Steven M. Management of the airway and lung isolation for thoracic surgery during the COVID-19 pandemic: Recommendations for clinical practice endorsed by the Association for Cardiothoracic Anaesthesia and Critical Care and the Society for Cardiothoracic Surgery in Great Britain and Ireland. *Anaesthesia*. May 5 2020;doi:10.1111/anae.15112

74. Raghavan R, Middleton PR, Mehdi A. Minimising aerosol generation during orthopaedic surgical procedures- Current practice to protect theatre staff during Covid-19 pandemic. *J Clin Orthop Trauma*. May-Jun 2020;11(3):506-507. doi:10.1016/j.jcot.2020.04.024

75. Mowbray NG, Ansell J, Horwood J, et al. Safe management of surgical smoke in the age of COVID-19. *Br J Surg*. May 3 2020;doi:10.1002/bjs.11679

76. David AP, Jiam NT, Reither JM, Gurrola JG, 2nd, Aghi MK, El-Sayed IH. Endoscopic skull base and transoral surgery during COVID-19 pandemic: Minimizing droplet spread with negative-pressure otolaryngology viral isolation drape. *Head Neck*. Jul 2020;42(7):1577-1582. doi:10.1002/hed.26239

77. Khan MM, Parab SR. Safety Guidelines for Sterility of Face Shields During COVID 19 Pandemic. *Indian J Otolaryngol Head Neck Surg*. Apr 30 2020:1-2. doi:10.1007/s12070-020-01865-2

78. Kampf G, Scheithauer S, Lemmen S, Saliou P, Suchomel M. COVID-19-associated shortage of alcohol-based hand rubs, face masks, medical gloves and gowns - proposal for a risk-adapted approach to ensure patient and healthcare worker safety. *J Hosp Infect*. Apr 29 2020;doi:10.1016/j.jhin.2020.04.041

79. Grossman J, Pierce A, Mody J, et al. Institution of a Novel Process for N95 Respirator Disinfection with Vaporized Hydrogen Peroxide in the Setting of the COVID-19 Pandemic at a Large Academic Medical Center. *J Am Coll Surg*. Aug 2020;231(2):275-280. doi:10.1016/j.jamcollsurg.2020.04.029

80. Garcia Godoy LR, Jones AE, Anderson TN, et al. Facial protection for healthcare workers during pandemics: a scoping review. *BMJ Glob Health*. May 2020;5(5)doi:10.1136/bmjgh-2020-002553

81. Thaler M, Khosravi I, Hirschmann MT, et al. Disruption of joint arthroplasty services in Europe during the COVID-19 pandemic: an online survey within the European Hip Society (EHS) and the European Knee Associates (EKA). *Knee Surg Sports Traumatol Arthrosc*. Jun 2020;28(6):1712-1719. doi:10.1007/s00167-020-06033-1

82. Liebensteiner MC, Khosravi I, Hirschmann MT, et al. Massive cutback in orthopaedic healthcare services due to the COVID-19 pandemic. *Knee Surg Sports Traumatol Arthrosc*. Jun 2020;28(6):1705-1711. doi:10.1007/s00167-020-06032-2

83. Ozturk CN, Kuruoglu D, Ozturk C, Rampazzo A, Gurunian Gurunluoglu R. Plastic Surgery and the COVID-19 Pandemic: A Review of Clinical Guidelines. *Ann Plast Surg*. Aug 2020;85(2S Suppl 2):S155-S160. doi:10.1097/SAP.0000000000002443

84. Civantos FJ, Leibowitz JM, Arnold DJ, et al. Ethical surgical triage of patients with head and neck cancer during the COVID-19 pandemic. *Head Neck*. Jul 2020;42(7):1423-1447. doi:10.1002/hed.26229

85. Coons BE, Tam SF, Okochi S. Rapid Development of Resident-Led Procedural Response Teams to Support Patient Care During the Coronavirus Disease 2019 Epidemic: A Surgical Workforce Activation Team. *JAMA Surg*. Apr 30 2020;doi:10.1001/jamasurg.2020.1782

86. Boccalatte LA, Larranaga JJ, Perez Raffo GM, et al. Brief guideline for the prevention of COVID-19 infection in head and neck and otolaryngology surgeons. *Am J Otolaryngol*. May - Jun 2020;41(3):102484. doi:10.1016/j.amjoto.2020.102484

87. De Simone B, Chouillard E, Di Saverio S, et al. Emergency surgery during the COVID-19 pandemic: what you need to know for practice. *Ann R Coll Surg Engl*. May 2020;102(5):323-332. doi:10.1308/rcsann.2020.0097

88. Germano A, Raffa G, Angileri FF, Cardali SM, Tomasello F. Coronavirus Disease 2019 (COVID-19) and Neurosurgery: Literature and Neurosurgical Societies Recommendations Update. *World Neurosurg*. Jul 2020;139:e812-e817. doi:10.1016/j.wneu.2020.04.181

89. Hojaij FC, Chinelatto LA, Boog GHP, Kasmirski JA, Lopes JVZ, Sacramento FM. Surgical Practice in the Current COVID-19 Pandemic: A Rapid Systematic Review. *Clinics (Sao Paulo)*. 2020;75:e1923. doi:10.6061/clinics/2020/e1923

90. Moletta L, Pierobon ES, Capovilla G, et al. International guidelines and recommendations for surgery during Covid-19 pandemic: A Systematic Review. *Int J Surg*. Jul 2020;79:180-188. doi:10.1016/j.ijsu.2020.05.061

91. Puliatti S, Eissa A, Eissa R, et al. COVID-19 and urology: a comprehensive review of the literature. *BJU Int*. Jun 2020;125(6):E7-E14. doi:10.1111/bju.15071

92. Soreide K, Hallet J, Matthews JB, et al. Immediate and long-term impact of the COVID-19 pandemic on delivery of surgical services. *Br J Surg*. Apr 30 2020;doi:10.1002/bjs.11670

93. Spock T, Kessler R, Lerner D, et al. Endoscopic Skull Base Surgery Protocol From the Frontlines: Transnasal Surgery During the COVID-19 Pandemic. *Otolaryngol Head Neck Surg*. May 26 2020:194599820931836. doi:10.1177/0194599820931836

94. Welsh Surgical Research Initiative Collaborative. Recommended operating room practice during the COVID-19 pandemic: systematic review. *BJS Open*. May 12 2020;doi:10.1002/bjs5.50304

95. Welsh Surgical Research Initiative Collaborative. Surgery during the COVID-19 pandemic: operating room suggestions from an international Delphi process. *Br J Surg*. May 12 2020;doi:10.1002/bjs.11747

96. Chen JX, Workman AD, Chari DA, et al. Demonstration and mitigation of aerosol and particle dispersion during mastoidectomy relevant to the COVID-19 era. *Otol Neurotol*. May 8 2020;doi:10.1097/MAO.0000000000002765

97. Howard BE. High-Risk Aerosol-Generating Procedures in COVID-19: Respiratory Protective Equipment Considerations. *Otolaryngol Head Neck Surg*. Jul 2020;163(1):98-103. doi:10.1177/0194599820927335

98. MacLeod C, Wilson P, Watson AJM. Colon capsule endoscopy: an innovative method for detecting colorectal pathology during the COVID-19 pandemic? *Colorectal Dis*. Jun 2020;22(6):621-624. doi:10.1111/codi.15134

99. Porter J, Blau E, Gharagozloo F, et al. Society of Robotic Surgery review: recommendations regarding the risk of COVID-19 transmission during minimally invasive surgery. *BJU Int*. Aug 2020;126(2):225-234. doi:10.1111/bju.15105

100. Thamboo A, Lea J, Sommer DD, et al. Clinical evidence based review and recommendations of aerosol generating medical procedures in otolaryngology - head and neck surgery during the COVID-19 pandemic. *J Otolaryngol Head Neck Surg*. May 6 2020;49(1):28. doi:10.1186/s40463-020-00425-6

101. Jessop ZM, Dobbs TD, Ali SR, et al. Personal Protective Equipment (PPE) for Surgeons during COVID-19 Pandemic: A Systematic Review of Availability, Usage, and Rationing. *Br J Surg*. May 12 2020;doi:10.1002/bjs.11750

102. Lam UN, Md Mydin Siddik NSF, Mohd Yussof SJ, Ibrahim S. N95 respirator associated pressure ulcer amongst COVID-19 health care workers. *Int Wound J*. May 12 2020;doi:10.1111/iwj.13398

103. Sobel D, Gn M, O'Rourke TK, Jr., et al. Personal Protective Equipment for Common Urologic Procedures Before and During the United States COVID-19 Pandemic: A Single Institution Study. *Urology*. Jul 2020;141:1-6. doi:10.1016/j.urology.2020.04.083

104. COVIDSurg Collaborative. Elective surgery cancellations due to the COVID-19 pandemic: global predictive modelling to inform surgical recovery plans. *Br J Surg*. May 12 2020;doi:10.1002/bjs.11746

105. Antonio Maria G, Vasileios P, Giacomo Piero I, et al. Urologic surgery and invasive procedures during coronavirus pandemic: Retrospective comparison of risk infection in a referral Covid hospital and in a free-Covid hospital. *Urologia*. May 10 2020:391560320927106. doi:10.1177/0391560320927106

106. Bedard NA, Elkins JM, Brown TS. Effect of COVID-19 on Hip and Knee Arthroplasty Surgical Volume in the United States. *J Arthroplasty*. Jul 2020;35(7S):S45-S48. doi:10.1016/j.arth.2020.04.060

107. Brown TS, Bedard NA, Rojas EO, et al. The Effect of the COVID-19 Pandemic on Electively Scheduled Hip and Knee Arthroplasty Patients in the United States. *J Arthroplasty*. Jul 2020;35(7S):S49-S55. doi:10.1016/j.arth.2020.04.052

108. Meyer M, Prost S, Farah K, et al. Spine Surgical Procedures during Coronavirus Disease 2019 Pandemic: Is It Still Possible to Take Care of Patients? Results of an Observational Study in the First Month of Confinement. *Asian Spine J*. Jun 2020;14(3):336-340. doi:10.31616/asj.2020.0197

109. Morlacco A, Motterle G, Zattoni F. The multifaceted long-term effects of the COVID-19 pandemic on urology. *Nat Rev Urol*. Jul 2020;17(7):365-367. doi:10.1038/s41585-020-0331-y

110. Ribal MJ, Cornford P, Briganti A, et al. European Association of Urology Guidelines Office Rapid Reaction Group: An Organisation-wide Collaborative Effort to Adapt the European Association of Urology Guidelines Recommendations to the Coronavirus Disease 2019 Era. *Eur Urol*. Jul 2020;78(1):21-28. doi:10.1016/j.eururo.2020.04.056

111. Wilson MP, Jack AS. Coronavirus disease 2019 (COVID-19) in neurology and neurosurgery: A scoping review of the early literature. *Clin Neurol Neurosurg*. Jun 2020;193:105866. doi:10.1016/j.clineuro.2020.105866

112. de Leeuw RA, Burger NB, Ceccaroni M, et al. COVID-19 and Laparoscopic Surgery: Scoping Review of Current Literature and Local Expertise. *JMIR Public Health Surveill*. Jun 23 2020;6(2):e18928. doi:10.2196/18928

113. Malik JS, Jenner C, Ward PA. Maximising application of the aerosol box in protecting healthcare workers during the COVID-19 pandemic. *Anaesthesia*. Jul 2020;75(7):974-975. doi:10.1111/anae.15109

114. Sharma D, Rubel KE, Ye MJ, et al. Cadaveric Simulation of Otologic Procedures: An Analysis of Droplet Splatter Patterns During the COVID-19 Pandemic. *Otolaryngol Head Neck Surg*. Aug 2020;163(2):320-324. doi:10.1177/0194599820930245

115. Sharma D, Rubel KE, Ye MJ, et al. Cadaveric Simulation of Endoscopic Endonasal Procedures: Analysis of Droplet Splatter Patterns During the COVID-19 Pandemic. *Otolaryngol Head Neck Surg*. Jul 2020;163(1):145-150. doi:10.1177/0194599820929274

116. Basso T, Dale H, Langvatn H, et al. Virus transmission during orthopedic surgery on patients with COVID-19 - a brief narrative review. *Acta Orthop*. May 14 2020:1-4. doi:10.1080/17453674.2020.1764234

117. Lescanne E, van der Mee-Marquet N, Juvanon JM, et al. Best practice recommendations: ENT consultations during the COVID-19 pandemic. *Eur Ann Otorhinolaryngol Head Neck Dis*. May 15 2020;doi:10.1016/j.anorl.2020.05.007

118. Mahadev S, Aroniadis OS, Barraza L, et al. Impact of the COVID-19 pandemic on endoscopy practice: results of a cross-sectional survey from the New York metropolitan area. *Gastrointest Endosc*. Apr 25 2020;doi:10.1016/j.gie.2020.04.047

119. Stewart CL, Thornblade LW, Diamond DJ, Fong Y, Melstrom LG. Personal Protective Equipment and COVID-19: A Review for Surgeons. *Ann Surg*. Aug 2020;272(2):e132-e138. doi:10.1097/SLA.0000000000003991

120. Torres AE, Lyons AB, Narla S, et al. Ultraviolet-C and other methods of decontamination of filtering facepiece N-95 respirators during the COVID-19 pandemic. *Photochem Photobiol Sci*. May 15 2020;doi:10.1039/d0pp00131g

121. Wong P, Kim Ong SG, Lim WY. COVID-19 and cardiopulmonary resuscitation: the recommended N95 mask may not be adequate. *Br J Anaesth*. May 18 2020;doi:10.1016/j.bja.2020.05.008

122. Armellini E, Repici A, Alvisi C, et al. Analysis of patients attitude to undergo urgent endoscopic procedures during COVID-19 outbreak in Italy. *Dig Liver Dis*. Jul 2020;52(7):695-699. doi:10.1016/j.dld.2020.05.015

123. Brindle ME, Gawande A. Managing COVID-19 in Surgical Systems. *Ann Surg*. Jul 2020;272(1):e1-e2. doi:10.1097/SLA.0000000000003923

124. Hulsbergen AFC, Eijkholt MM, Balak N, et al. Ethical triage during the COVID-19 pandemic: a toolkit for neurosurgical resource allocation. *Acta Neurochir (Wien)*. Jul 2020;162(7):1485-1490. doi:10.1007/s00701-020-04375-w

125. Kaye K, Paprottka F, Escudero R, et al. Elective, Non-urgent Procedures and Aesthetic Surgery in the Wake of SARS-COVID-19: Considerations Regarding Safety, Feasibility and Impact on Clinical Management. *Aesthetic Plast Surg*. Jun 2020;44(3):1014-1042. doi:10.1007/s00266-020-01752-9

126. Michel F, Gaillet S, Cornu JN, et al. [French Association of Urology. COVID-19: Recommendations for functional urology]. *Prog Urol*. Jun - Jul 2020;30(8-9):414-425. Recommandations jointes des comites de l'AFU (neuro-urologie, CUROPF, CTMH) pour l'urologie fonctionnelle durant la crise sanitaire liee au COVID-19. doi:10.1016/j.purol.2020.04.007

127. Mayo-Yanez M, Calvo-Henriquez C, Lechien JR, Fakhry N, Ayad T, Chiesa-Estomba CM. Is the ultrasonic scalpel recommended in head and neck surgery during the COVID-19 pandemic? State-of-the-art review. *Head Neck*. Jul 2020;42(7):1657-1663. doi:10.1002/hed.26278

128. Mintz Y, Arezzo A, Boni L, et al. The risk of COVID-19 transmission by laparoscopic smoke may be lower than for laparotomy: a narrative review. *Surg Endosc*. Aug 2020;34(8):3298-3305. doi:10.1007/s00464-020-07652-y

129. Ruiz-Manriquez J, Leon-Lara X, Campos-Murguia A, et al. Knowledge of Latin American gastroenterologists and endoscopists regarding SARS-CoV-2 infection. *Rev Gastroenterol Mex*. Jul - Sep 2020;85(3):288-294. Conocimiento sobre la infeccion por SARS-CoV-2 de Gastroenterologos y Endoscopistas de Latino America. doi:10.1016/j.rgmx.2020.04.003

130. Workman AD, Jafari A, Welling DB, et al. Airborne Aerosol Generation During Endonasal Procedures in the Era of COVID-19: Risks and Recommendations. *Otolaryngol Head Neck Surg*. May 26 2020:194599820931805. doi:10.1177/0194599820931805

131. Chan JF, Yuan S, Zhang AJ, et al. Surgical mask partition reduces the risk of non-contact transmission in a golden Syrian hamster model for Coronavirus Disease 2019 (COVID-19). *Clin Infect Dis*. May 30 2020;doi:10.1093/cid/ciaa644

132. Desborough J, Hall Dykgraaf S, Rankin D, Kidd M. The importance of consistent advice during a pandemic: An analysis of Australian advice regarding personal protective equipment in healthcare settings during COVID-19. *Aust J Gen Pract*. Jun 2020;49(6):369-372. doi:10.31128/AJGP-04-20-5374

133. Kumar J, Katto MS, Siddiqui AA, et al. Knowledge, Attitude, and Practices of Healthcare Workers Regarding the Use of Face Mask to Limit the Spread of the New Coronavirus Disease (COVID-19). *Cureus*. Apr 20 2020;12(4):e7737. doi:10.7759/cureus.7737

134. Li KKW, Joussen AM, Kwan JKC, Steel DHW. FFP3, FFP2, N95, surgical masks and respirators: what should we be wearing for ophthalmic surgery in the COVID-19 pandemic? *Graefes Arch Clin Exp Ophthalmol*. Aug 2020;258(8):1587-1589. doi:10.1007/s00417-020-04751-3

135. Torzilli G, Vigano L, Galvanin J, et al. A Snapshot of Elective Oncological Surgery in Italy During COVID-19 Emergency: Pearls, Pitfalls, and Perspectives. *Ann Surg*. Aug 2020;272(2):e112-e117. doi:10.1097/SLA.0000000000004081

136. Antony J, James WT, Neriamparambil AJ, Barot DD, Withers T. An Australian Response to the COVID-19 Pandemic and Its Implications on the Practice of Neurosurgery. *World Neurosurg*. Jul 2020;139:e864-e871. doi:10.1016/j.wneu.2020.05.136

137. Baud G, Brunaud L, Lifante JC, et al. Endocrine surgery during and after the COVID-19 epidemic: Expert guidelines from AFCE. *J Visc Surg*. Jun 2020;157(3S1):S43-S49. doi:10.1016/j.jviscsurg.2020.04.018

138. Bilal M, Simons M, Rahman AU, et al. What constitutes urgent endoscopy? A social media snapshot of gastroenterologists' views during the COVID-19 pandemic. *Endosc Int Open*. May 2020;8(5):E693-E698. doi:10.1055/a-1153-9014

139. Hemingway JF, Singh N, Starnes BW. Emerging practice patterns in vascular surgery during the COVID-19 pandemic. *J Vasc Surg*. Aug 2020;72(2):396-402. doi:10.1016/j.jvs.2020.04.492

140. La Torre M, Pata F, Gallo G. Delayed benign surgery during the COVID-19 pandemic: the other side of the coin. *Br J Surg*. Jul 2020;107(8):e258. doi:10.1002/bjs.11712

141. Latz CA, Boitano LT, Png CYM, et al. Early vascular surgery response to the COVID-19 pandemic: Results of a nationwide survey. *J Vasc Surg*. May 23 2020;doi:10.1016/j.jvs.2020.05.032

142. Patriti A, Baiocchi GL, Catena F, Marini P, Catarci M, Italiani FobotACO. Emergency general surgery in Italy during the COVID-19 outbreak: first survey from the real life. *World J Emerg Surg*. May 24 2020;15(1):36. doi:10.1186/s13017-020-00314-3

143. Pini Prato A, Conforti A, Almstrom M, et al. Management of COVID-19-Positive Pediatric Patients Undergoing Minimally Invasive Surgical Procedures: Systematic Review and Recommendations of the Board of European Society of Pediatric Endoscopic Surgeons. *Front Pediatr*. 2020;8:259. doi:10.3389/fped.2020.00259

144. Shen Y, Cheng CS, Wang P, et al. CSCO ablation expert workshop report: Recommendations for the management of tumor ablation during the coronavirus disease 2019 epidemic. *J Cancer Res Ther*. 2020;16(2):350-355. doi:10.4103/jcrt.JCRT_480_20

145. Chu DK, Akl EA, Duda S, et al. Physical distancing, face masks, and eye protection to prevent person-to-person transmission of SARS-CoV-2 and COVID-19: a systematic review and meta-analysis. *Lancet*. Jun 27 2020;395(10242):1973-1987. doi:10.1016/S0140-6736(20)31142-9

146. Kuhar HN, Heilingoetter A, Bergman M, Worobetz N, Chiang T, Matrka L. Otolaryngology in the Time of Corona: Assessing Operative Impact and Risk During the COVID-19 Crisis. *Otolaryngol Head Neck Surg*. Aug 2020;163(2):307-315. doi:10.1177/0194599820930214

147. Maida M, Sferrazza S, Savarino E, et al. Impact of the COVID-19 pandemic on Gastroenterology Divisions in Italy: A national survey. *Dig Liver Dis*. Aug 2020;52(8):808-815. doi:10.1016/j.dld.2020.05.017

148. Ozoner B, Gungor A, Hasanov T, Toktas ZO, Kilic T. Neurosurgical Practice During Coronavirus Disease 2019 (COVID-19) Pandemic. *World Neurosurg*. Aug 2020;140:198-207. doi:10.1016/j.wneu.2020.05.195

149. Supehia S, Singh V, Sharma T, Khapre M, Gupta PK. Rational use of face mask in a tertiary care hospital setting during COVID-19 pandemic: An observational study. *Indian J Public Health*. Jun 2020;64(Supplement):S225-S227. doi:10.4103/ijph.IJPH_493_20

150. Szarpak L, Smereka J, Filipiak KJ, Ladny JR, Jaguszewski M. Cloth masks versus medical masks for COVID-19 protection. *Cardiol J*. 2020;27(2):218-219. doi:10.5603/CJ.a2020.0054

151. Gupta P, Muthukumar N, Rajshekhar V, et al. Neurosurgery and Neurology Practices during the Novel COVID-19 Pandemic: A Consensus Statement from India. *Neurol India*. Mar-Apr 2020;68(2):246-254. doi:10.4103/0028-3886.283130

152. Gravas S, Bolton D, Gomez R, et al. Impact of COVID-19 on Urology Practice: A Global Perspective and Snapshot Analysis. *J Clin Med*. Jun 3 2020;9(6)doi:10.3390/jcm9061730

153. Kamarajah SK, Markar SR, Singh P, Griffiths EA, Oesophagogastric Anastomosis Audit G. The influence of the SARS-CoV-2 pandemic on esophagogastric cancer services: an international survey of esophagogastric surgeons. *Dis Esophagus*. Jun 5 2020;doi:10.1093/dote/doaa054

154. Laux CJ, Bauer DE, Kohler A, Uckay I, Farshad M. Disproportionate Case Reduction After Ban of Elective Surgeries During the SARS-CoV-2 Pandemic. *Clin Spine Surg*. Jul 2020;33(6):244-246. doi:10.1097/BSD.0000000000001017

155. Waledziak M, Rozanska-Waledziak A, Pedziwiatr M, et al. Bariatric Surgery During COVID-19 Pandemic from Patients' Point of View-The Results of a National Survey. *J Clin Med*. Jun 2 2020;9(6)doi:10.3390/jcm9061697

156. Daigle P, Leung V, Yin V, Kalin-Hajdu E, Nijhawan N. Personal protective equipment (PPE) during the COVID-19 pandemic for oculofacial plastic and orbital surgery. *Orbit*. Jun 18 2020:1-6. doi:10.1080/01676830.2020.1781200

157. Heldwein FL, Loeb S, Wroclawski ML, et al. A Systematic Review on Guidelines and Recommendations for Urology Standard of Care During the COVID-19 Pandemic. *Eur Urol Focus*. Sep 15 2020;6(5):1070-1085. doi:10.1016/j.euf.2020.05.020

158. Krajewska Wojciechowska J, Krajewski W, Zub K, Zatonski T. Review of practical recommendations for otolaryngologists and head and neck surgeons during the COVID-19 pandemic. *Auris Nasus Larynx*. Jun 6 2020;doi:10.1016/j.anl.2020.05.022

159. Lagos AE, Ramos PH, Andrade T. Protection for Otolaryngologic Surgery in the COVID-19 Pandemic. *OTO Open*. Apr-Jun 2020;4(2):2473974X20934734. doi:10.1177/2473974X20934734

160. Shokri T, Saadi RA, Liaw J, et al. Facial Plastic and Reconstructive Surgery During the COVID-19 Pandemic: Implications in Craniomaxillofacial Trauma and Head and Neck Reconstruction. *Ann Plast Surg*. Aug 2020;85(2S Suppl 2):S166-S170. doi:10.1097/SAP.0000000000002492

161. Viswanathan VK, Subramanian S, Rao AK. Principles for Managing Patients with Spinal Ailments in the Coronavirus Disease 2019 Era: What Do We Know So Far? An Evidence-Based, Narrative Review. *Asian Spine J*. Aug 2020;14(4):572-580. doi:10.31616/asj.2020.0248

162. Wang Y, Zeng L, Yao S, et al. Recommendations of protective measures for orthopedic surgeons during COVID-19 pandemic. *Knee Surg Sports Traumatol Arthrosc*. Jul 2020;28(7):2027-2035. doi:10.1007/s00167-020-06092-4

163. Begley JL, Lavery KE, Nickson CP, Brewster DJ. The aerosol box for intubation in coronavirus disease 2019 patients: an in-situ simulation crossover study. *Anaesthesia*. Aug 2020;75(8):1014-1021. doi:10.1111/anae.15115

164. Campanile FC, Podda M, Arezzo A, et al. Acute cholecystitis during COVID-19 pandemic: a multisocietary position statement. *World J Emerg Surg*. Jun 8 2020;15(1):38. doi:10.1186/s13017-020-00317-0

165. Cheng VC, Wong SC, Chan VW, et al. Air and environmental sampling for SARS-CoV-2 around hospitalized patients with coronavirus disease 2019 (COVID-19). *Infect Control Hosp Epidemiol*. Jun 8 2020:1-8. doi:10.1017/ice.2020.282

166. Matava CT, Yu J, Denning S. Clear plastic drapes may be effective at limiting aerosolization and droplet spray during extubation: implications for COVID-19. *Can J Anaesth*. Jul 2020;67(7):902-904. doi:10.1007/s12630-020-01649-w

167. Pavan N, Crestani A, Abrate A, et al. Risk of Virus Contamination Through Surgical Smoke During Minimally Invasive Surgery: A Systematic Review of the Literature on a Neglected Issue Revived in the COVID-19 Pandemic Era. *Eur Urol Focus*. Sep 15 2020;6(5):1058-1069. doi:10.1016/j.euf.2020.05.021

168. Liu M, Cheng SZ, Xu KW, et al. Use of personal protective equipment against coronavirus disease 2019 by healthcare professionals in Wuhan, China: cross sectional study. *BMJ*. Jun 10 2020;369:m2195. doi:10.1136/bmj.m2195

169. MacIntyre CR, Chughtai AA. A rapid systematic review of the efficacy of face masks and respirators against coronaviruses and other respiratory transmissible viruses for the community, healthcare workers and sick patients. *Int J Nurs Stud*. Aug 2020;108:103629. doi:10.1016/j.ijnurstu.2020.103629

170. Paderno A, Fior M, Berretti G, et al. SARS-CoV-2 Infection in Health Care Workers: Cross-sectional Analysis of an Otolaryngology Unit. *Otolaryngol Head Neck Surg*. Jun 2 2020:194599820932162. doi:10.1177/0194599820932162

171. Parush A, Wacht O, Gomes R, Frenkel A. Human Factor Considerations in Using Personal Protective Equipment in the COVID-19 Pandemic Context: Binational Survey Study. *J Med Internet Res*. Jun 17 2020;22(6):e19947. doi:10.2196/19947

172. Mazzatenta D, Zoli M, Cavallo MA, et al. Remodulation of neurosurgical activities in an Italian region (Emilia-Romagna) under COVID- 19 emergency: maintaining the standard of care during the crisis. *J Neurosurg Sci*. Jun 11 2020;doi:10.23736/S0390-5616.20.05018-3

173. Zakka K, Erridge S, Chidambaram S, et al. Electrocautery, Diathermy, and Surgical Energy Devices: Are Surgical Teams at Risk During the COVID-19 Pandemic? *Ann Surg*. Jun 9 2020;doi:10.1097/SLA.0000000000004112

174. Qaseem A, Etxeandia-Ikobaltzeta I, Yost J, et al. Use of N95, Surgical, and Cloth Masks to Prevent COVID-19 in Health Care and Community Settings: Living Practice Points From the American College of Physicians (Version 1). *Ann Intern Med*. Jun 18 2020;doi:10.7326/M20-3234

175. Crespo J, Andrade R, Alberca de Las Parras F, et al. Resumption of activity in gastroenterology departments. Recommendations by SEPD, AEEH, GETECCU and AEG. *Rev Esp Enferm Dig*. May 2020;112(5):397-411. doi:10.17235/reed.2020.7141/2020

176. DeFazio JR, Kahan A, Fallon EM, et al. Development of pediatric surgical decision-making guidelines for COVID-19 in a New York City children's hospital. *J Pediatr Surg*. Aug 2020;55(8):1427-1430. doi:10.1016/j.jpedsurg.2020.05.043

177. Ingram ME, Raval MV, Newton C, Lopez ME, Berman L. Characterization of initial North American pediatric surgical response to the COVID-19 pandemic. *J Pediatr Surg*. Aug 2020;55(8):1431-1435. doi:10.1016/j.jpedsurg.2020.06.001

178. Silva LED, Cohen RV, JC DE-A, et al. The recommendations of the Brazilian College of Surgeons and the Brazilian Bariatric and Metabolic Surgery Societies on the return of bariatric and metabolic operations in geographic regions of the country where the procedures have been allowed by local policies, in the period of COVID-19 pandemic. *Rev Col Bras Cir*. 2020;47:e20202640. doi:10.1590/0100-6991e-20202640

179. Darcy K, Elhaddad O, Achiron A, et al. Reducing visible aerosol generation during phacoemulsification in the era of Covid-19. *Eye (Lond)*. Jun 26 2020;doi:10.1038/s41433-020-1053-3

180. Liyanage S, Ramasamy P, Elhaddad O, Darcy K, Hudson A, Keller J. Assessing visible aerosol generation during vitrectomy in the era of Covid-19. *Eye (Lond)*. Jun 25 2020;doi:10.1038/s41433-020-1052-4

181. Safari S, Keyvani H, Alamdari NM, et al. Abdominal Surgery in Patients with COVID-19: Detection of SARS-CoV-2 in Abdominal and Adipose Tissues. *Ann Surg*. Jun 16 2020;doi:10.1097/SLA.0000000000004165

182. Chou R, Dana T, Jungbauer R, Weeks C, McDonagh MS. Masks for Prevention of Respiratory Virus Infections, Including SARS-CoV-2, in Health Care and Community Settings: A Living Rapid Review. *Ann Intern Med*. Jun 24 2020;doi:10.7326/M20-3213

183. Jones P, Roberts S, Hotu C, Kamona S. What proportion of healthcare worker masks carry virus? A systematic review. *Emerg Med Australas*. Jun 24 2020;doi:10.1111/1742-6723.13581

184. Zangmeister CD, Radney JG, Vicenzi EP, Weaver JL. Filtration Efficiencies of Nanoscale Aerosol by Cloth Mask Materials Used to Slow the Spread of SARS-CoV-2. *ACS Nano*. Jul 28 2020;14(7):9188-9200. doi:10.1021/acsnano.0c05025

185. Anteby R, Zager Y, Barash Y, et al. The Impact of the Coronavirus Disease 2019 Outbreak on the Attendance of Patients with Surgical Complaints at a Tertiary Hospital Emergency Department. *J Laparoendosc Adv Surg Tech A*. Jun 23 2020;doi:10.1089/lap.2020.0465

186. Bonalumi G, Giambuzzi I, Barbone A, et al. A call to action becomes practice: cardiac and vascular surgery during the COVID-19 pandemic based on the Lombardy emergency guidelines. *Eur J Cardiothorac Surg*. Aug 1 2020;58(2):319-327. doi:10.1093/ejcts/ezaa204

187. Gravas S, Fournier G, Oya M, et al. Prioritising Urological Surgery in the COVID-19 Era: A Global Reflection on Guidelines. *Eur Urol Focus*. Sep 15 2020;6(5):1104-1110. doi:10.1016/j.euf.2020.06.006

188. Papalia R, Cataldo R, Alloni R, et al. Urologic surgery in a safe hospital during the COVID-19 pandemic scenario. *Minerva Urol Nefrol*. Jun 22 2020;doi:10.23736/S0393-2249.20.03923-5

189. Tivey DR, Davis SS, Kovoor JG, et al. Safe surgery during the coronavirus disease 2019 crisis. *ANZ J Surg*. Jun 28 2020;doi:10.1111/ans.16089

190. Couto RA, Wiener TC, Adams WP. Evaluating Postoperative Outcomes of Patients Undergoing Elective Procedures in an Ambulatory Surgery Center During the COVID-19 Pandemic. *Aesthet Surg J*. Jun 29 2020;doi:10.1093/asj/sjaa180

191. Gadkaree SK, Derakhshan A, Workman AD, Feng AL, Quesnel AM, Shaye DA. Quantifying Aerosolization of Facial Plastic Surgery Procedures in the COVID-19 Era: Safety and Particle Generation in Craniomaxillofacial Trauma and Rhinoplasty. *Facial Plast Surg Aesthet Med*. Jul 2 2020;doi:10.1089/fpsam.2020.0322

192. Anderegg L, Meisenhelder C, Ngooi CO, et al. A scalable method of applying heat and humidity for decontamination of N95 respirators during the COVID-19 crisis. *PLoS One*. 2020;15(7):e0234851. doi:10.1371/journal.pone.0234851

193. Grinshpun SA, Yermakov M, Khodoun M. Autoclave sterilization and ethanol treatment of re-used surgical masks and N95 respirators during COVID-19: impact on their performance and integrity. *J Hosp Infect*. Aug 2020;105(4):608-614. doi:10.1016/j.jhin.2020.06.030

194. Lockhart SL, Naidu JJ, Badh CS, Duggan LV. Simulation as a tool for assessing and evolving your current personal protective equipment: lessons learned during the coronavirus disease (COVID-19) pandemic. *Can J Anaesth*. Jul 2020;67(7):895-896. doi:10.1007/s12630-020-01638-z

195. Sinonquel P, Aerts M, Badaoui A, et al. BSGIE survey on COVID-19 and gastrointestinal endoscopy in Belgium : results and recommendations. *Acta Gastroenterol Belg*. Apr-Jun 2020;83(2):344-354.

196. Balakrishnan A, Lesurtel M, Siriwardena AK, et al. Delivery of hepato-pancreato-biliary surgery during the COVID-19 pandemic: an European-African Hepato-Pancreato-Biliary Association (E-AHPBA) cross-sectional survey. *HPB (Oxford)*. Aug 2020;22(8):1128-1134. doi:10.1016/j.hpb.2020.05.012

197. Gironi LC, Boggio P, Giorgione R, et al. The impact of COVID-19 pandemics on dermatologic surgery: real-life data from the Italian Red-Zone. *J Dermatolog Treat*. Jul 7 2020:1-7. doi:10.1080/09546634.2020.1789044

198. Arantes VN, Martins BC, Seqatto R, et al. Impact of coronavirus pandemic crisis in endoscopic clinical practice: Results from a national survey in Brazil. *Endosc Int Open*. Jun 2020;8(6):E822-E829. doi:10.1055/a-1183-3324

199. Pandey AS, Ringer AJ, Rai AT, et al. Minimizing SARS-CoV-2 exposure when performing surgical interventions during the COVID-19 pandemic. *J Neurointerv Surg*. Jul 2020;12(7):643-647. doi:10.1136/neurintsurg-2020-016161

200. Barratt R, Shaban RZ, Gilbert GL. Characteristics of personal protective equipment training programs in Australia and New Zealand hospitals: A survey. *Infect Dis Health*. Jun 26 2020;doi:10.1016/j.idh.2020.05.005

201. Chandra A, Haynes R, Burdon M, et al. Personal protective equipment (PPE) for vitreoretinal surgery during COVID-19. *Eye (Lond)*. Jul 2020;34(7):1196-1199. doi:10.1038/s41433-020-0948-3

202. Jung J, Kim JY, Bae S, et al. Contamination of personal protective equipment by SARS-CoV-2 during routine care of patients with mild COVID-19. *J Infect*. Aug 2020;81(2):e165-e167. doi:10.1016/j.jinf.2020.06.021

203. Khattab MF, Kannan TMA, Morsi A, et al. The short-term impact of COVID-19 pandemic on spine surgeons: a cross-sectional global study. *Eur Spine J*. Aug 2020;29(8):1806-1812. doi:10.1007/s00586-020-06517-1

204. Pinheiro RN, Coimbra FJF, Costa-Jr WLD, et al. Surgical cancer care in the COVID-19 era: front line views and consensus. *Rev Col Bras Cir*. 2020;47:e20202601. doi:10.1590/0100-6991e-20202601

205. Sommerstein R, Fux CA, Vuichard-Gysin D, et al. Risk of SARS-CoV-2 transmission by aerosols, the rational use of masks, and protection of healthcare workers from COVID-19. *Antimicrob Resist Infect Control*. Jul 6 2020;9(1):100. doi:10.1186/s13756-020-00763-0

206. Tan GSE, Linn KZ, Soon MML, et al. Effect of extended use N95 respirators and eye protection on personal protective equipment (PPE) utilization during SARS-CoV-2 outbreak in Singapore. *Antimicrob Resist Infect Control*. Jun 15 2020;9(1):86. doi:10.1186/s13756-020-00753-2

207. Teh KKJ, Tay SW, Chen K, et al. Impact of enhanced personal protective equipment on colonoscopy performance during the COVID-19 pandemic. *Endosc Int Open*. Jun 2020;8(6):E809-E814. doi:10.1055/a-1167-1703

208. Britton CR, Hayman G, Macfarlane C, et al. COVID-19 preparedness and response at a large UK major trauma operating theatres department. *J Perioper Pract*. Jul 2020;30(7-8):210-220. doi:10.1177/1750458920934406

209. Mathiesen T, Arraez M, Asser T, et al. A snapshot of European neurosurgery December 2019 vs. March 2020: just before and during the Covid-19 pandemic. *Acta Neurochir (Wien)*. Sep 2020;162(9):2221-2233. doi:10.1007/s00701-020-04482-8

210. Jun ISY, Hui KKO, Songbo PZ. Perspectives on Coronavirus Disease 2019 Control Measures for Ophthalmology Clinics Based on a Singapore Center Experience. *JAMA Ophthalmol*. Mar 31 2020;doi:10.1001/jamaophthalmol.2020.1288

211. Rakovich G, Urbanowicz R, Issa R, Wang HT. Minimizing the Risk of Aerosol Contamination During Elective Lung Resection Surgery. *Ann Surg*. Aug 2020;272(2):e125-e128. doi:10.1097/SLA.0000000000004087

212. Tan L, Kovoor JG, Williamson P, et al. Personal Protective Equipment and Evidence-Based Advice for Surgical Departments during COVID-19. *ANZ J Surg*. Jul 15 2020;doi:10.1111/ans.16194

213. Arellano-Cotrina JJ, Marengo-Coronel N, Atoche-Socola KJ, Pena-Soto C, Arriola-Guillen LE. Effectiveness and Recommendations for the Use of Dental Masks in the Prevention of COVID-19: A Literature Review. *Disaster Med Public Health Prep*. Jul 17 2020:1-6. doi:10.1017/dmp.2020.255

214. Carnino JM, Ryu S, Ni K, Jin Y. Pretreated household materials carry similar filtration protection against pathogens when compared with surgical masks. *Am J Infect Control*. Aug 2020;48(8):883-889. doi:10.1016/j.ajic.2020.05.024

215. Furuta T, Irisawa A, Matsumoto T, et al. Clinical Questions and Answers on Gastrointestinal Endoscopy during the Novel COVID-19 Pandemic. *Dig Endosc*. May 29 2020;doi:10.1111/den.13757

216. O’Hearn K, Gertsman S, Sampson M, et al. Decontaminating N95 and SN95 masks with Ultraviolet Germicidal Irradiation (UVGI) does not impair mask efficacy and safety: A Systematic Review. *J Hosp Infect*. Jul 17 2020;doi:10.1016/j.jhin.2020.07.014

217. Sawhney MS, Bilal M, Pohl H, et al. Triaging advanced GI endoscopy procedures during the COVID-19 pandemic: consensus recommendations using the Delphi method. *Gastrointest Endosc*. May 16 2020;doi:10.1016/j.gie.2020.05.014

218. Babidge WJ, Tivey DR, Kovoor JG, et al. Surgery Triage during the COVID-19 Pandemic. *ANZ J Surg*. Jul 20 2020;doi:10.1111/ans.16196

219. Ding BTK, Tan KG, Oh JY, Lee KT. Orthopaedic surgery after COVID-19 - A blueprint for resuming elective surgery after a pandemic. *Int J Surg*. Aug 2020;80:162-167. doi:10.1016/j.ijsu.2020.07.012

220. Giuntoli M, Bonicoli E, Bugelli G, Valesini M, Manca M, Scaglione M. Lessons learnt from COVID 19: An Italian multicentric epidemiological study of orthopaedic and trauma services. *J Clin Orthop Trauma*. Jul-Aug 2020;11(4):721-727. doi:10.1016/j.jcot.2020.05.021

221. Haffer H, Schomig F, Rickert M, et al. Impact of the COVID-19 Pandemic on Orthopaedic and Trauma Surgery in University Hospitals in Germany: Results of a Nationwide Survey. *J Bone Joint Surg Am*. Jul 15 2020;102(14):e78. doi:10.2106/JBJS.20.00756

222. O'Leary MP, Choong KC, Thornblade LW, Fakih MG, Fong Y, Kaiser AM. Management Considerations for the Surgical Treatment of Colorectal Cancer During the Global Covid-19 Pandemic. *Ann Surg*. Aug 2020;272(2):e98-e105. doi:10.1097/SLA.0000000000004029

223. Christopherson DA, Yao WC, Lu M, Vijayakumar R, Sedaghat AR. High-Efficiency Particulate Air Filters in the Era of COVID-19: Function and Efficacy. *Otolaryngol Head Neck Surg*. Jul 14 2020:194599820941838. doi:10.1177/0194599820941838

224. da Costa KM, Saxena AK. Coronavirus disease 2019 pandemic and identifying insufflators with desufflation mode and surgical smoke evacuators for safe CO2 removal. *Asian J Endosc Surg*. Jul 26 2020;doi:10.1111/ases.12834

225. Druel T, Andeol Q, Rongieras F, Bertani A, Bordes M, Alvernhe A. Evaluation of containment measures' effect on orthopaedic trauma surgery during the COVID-19 pandemic: a retrospective comparison between 2019 and 2020. *Int Orthop*. Jul 21 2020;doi:10.1007/s00264-020-04712-9

226. Zhang J, Aslanides IM, Selimis V, et al. A Comprehensive Strategy for Laser Corneal Refractive Surgery during the COVID-19 Epidemic in a Tertiary Teaching Hospital in Wenzhou, China. *J Ophthalmol*. 2020;2020:4835630. doi:10.1155/2020/4835630

227. Steward JE, Kitley WR, Schmidt CM, Sundaram CP. Urologic Surgery and COVID-19: How the Pandemic Is Changing the Way We Operate. *J Endourol*. May 2020;34(5):541-549. doi:10.1089/end.2020.0342

228. Simpson A, Dall G, Haas JG. COVID-19: potential transmission through aerosols in surgical procedures and blood products. *Bone Joint Res*. Apr 2020;9(4):200-201. doi:10.1302/2046-3758.94.BJR-2020-0130

229. Patterson TJ, Currie PJ, Beck J, Spence RAJ, Spence GM. A systematic review of viral transmission risk to healthcare staff comparing laparoscopic and open surgery. *Surgeon*. Jul 17 2020;doi:10.1016/j.surge.2020.06.016

230. Ruggieri P, Trovarelli G, Angelini A, Pala E, Berizzi A, Donato D. COVID-19 strategy in organizing and planning orthopedic surgery in a major orthopedic referral center in an area of Italy severely affected by the pandemic: experience of the Department of Orthopedics, University of Padova. *J Orthop Surg Res*. Jul 23 2020;15(1):279. doi:10.1186/s13018-020-01740-4

231. Wilson AM, Abney SE, King MF, et al. COVID-19 and use of non-traditional masks: how do various materials compare in reducing the risk of infection for mask wearers? *J Hosp Infect*. Aug 2020;105(4):640-642. doi:10.1016/j.jhin.2020.05.036

232. Steinberg BE, Aoyama K, McVey M, et al. Efficacy and safety of decontamination for N95 respirator reuse: a systematic literature search and narrative synthesis. *Can J Anaesth*. Jul 27 2020;Efficacite et securite de la decontamination visant la reutilisation des masques N95 : recherche de litterature systematique et synthese narrative. doi:10.1007/s12630-020-01770-w

233. Seresirikachorn K, Phoophiboon V, Chobarporn T, et al. Decontamination and reuse of surgical masks and N95 filtering facepiece respirators during the COVID-19 pandemic: A systematic review. *Infect Control Hosp Epidemiol*. Jul 30 2020:1-6. doi:10.1017/ice.2020.379

234. Ren Y, Liu S, Yang L, et al. Practice and exploration of infection prevention and control measures based on risk management of surgical patients during the epidemic of corona virus disease 2019 (COVID-19). *Am J Infect Control*. Jul 21 2020;doi:10.1016/j.ajic.2020.07.023

235. Khan MA, Sivalingam A, Haller JA. Perceptions of Occupational Risk and Changes in Clinical Practice of United States Vitreoretinal Surgery Fellows during the COVID-19 Pandemic. *Ophthalmol Retina*. May 22 2020;doi:10.1016/j.oret.2020.05.011

236. Kempfle JS, Panda A, Hottin M, et al. Effect of Powered Air-Purifying Respirators on Speech Recognition Among Health Care Workers. *Otolaryngol Head Neck Surg*. Jul 21 2020:194599820945685. doi:10.1177/0194599820945685

237. Ahluwalia R, Rocque BG, Shannon CN, Blount JP. The impact of imposed delay in elective pediatric neurosurgery: an informed hierarchy of need in the time of mass casualty crisis. *Childs Nerv Syst*. Jul 2020;36(7):1347-1355. doi:10.1007/s00381-020-04671-x

238. Senent-Boza A, Benitez-Linero I, Tallon-Aguilar L, et al. Early implementation of protective measures defines surgical outcomes in the COVID-19 pandemic. *Surg Today*. Sep 2020;50(9):1107-1112. doi:10.1007/s00595-020-02080-w

239. Saibene AM, Allevi F, Biglioli F, Felisati G. Role and Management of a Head and Neck Department during the COVID-19 Outbreak in Lombardy. *Otolaryngol Head Neck Surg*. Jun 2020;162(6):795-796. doi:10.1177/0194599820917914

240. Panigrahi M, Kakani N, Vooturi S. Impact of SARS-Cov2 on Endoscopic Trans-Nasal Skull Base Surgeries. *Neurol India*. May-Jun 2020;68(Supplement):S141-S145. doi:10.4103/0028-3886.287683

241. Galli J, Settimi S, Tricarico L, Almadori G, Paludetti G. Clinical and surgical management of patients with head and neck cancer in a COVID-19 dedicated center in Italy. *Head Neck*. Jul 2020;42(7):1466-1470. doi:10.1002/hed.26263

242. Sowerby LJ, Stephenson K, Dickie A, et al. International Registry of Otolaryngologist - Head and Neck Surgeons with COVID-19. *Int Forum Allergy Rhinol*. Jul 31 2020;doi:10.1002/alr.22677

243. Zhang J, Sun M, Li N, et al. Acquired infection after intubating patients with COVID-19: A retrospective pilot study. *J Clin Anesth*. Jul 21 2020;67:110006. doi:10.1016/j.jclinane.2020.110006

244. Brun D, Curti C, Mekideche T, et al. Stockpiled N95 respirator/surgical mask release beyond manufacturer-designated shelf-life: a French experience. *J Hosp Infect*. Jul 31 2020;doi:10.1016/j.jhin.2020.07.032

245. Lam SC, Arora T, Grey I, et al. Perceived Risk and Protection From Infection and Depressive Symptoms Among Healthcare Workers in Mainland China and Hong Kong During COVID-19. *Front Psychiatry*. 2020;11:686. doi:10.3389/fpsyt.2020.00686

246. Prakash G, Shetty P, Thiagarajan S, et al. Compliance and perception about personal protective equipment among health care workers involved in the surgery of COVID-19 negative cancer patients during the pandemic. *J Surg Oncol*. Aug 3 2020;doi:10.1002/jso.26151

247. Scarano A, Inchingolo F, Lorusso F. Facial Skin Temperature and Discomfort When Wearing Protective Face Masks: Thermal Infrared Imaging Evaluation and Hands Moving the Mask. *Int J Environ Res Public Health*. Jun 27 2020;17(13)doi:10.3390/ijerph17134624

248. Bansal H, Karpe A, Mittal S, Trikha V. Amid COVID-19 pandemic-an innovative use of local resources to substitute the need for specialized operation theatre tables for orthopaedic and trauma surgery. *Int Orthop*. Aug 3 2020;doi:10.1007/s00264-020-04758-9

249. Gammeri E, Cillo GM, Sunthareswaran R, Magro T. Is a "COVID-19-free" hospital the answer to resuming elective surgery during the current pandemic? Results from the first available prospective study. *Surgery*. Jul 15 2020;doi:10.1016/j.surg.2020.07.003

250. Simonato A, Giannarini G, Abrate A, et al. Clinical pathways for urology patients during the COVID-19 pandemic. *Minerva Urol Nefrol*. Jun 2020;72(3):376-383. doi:10.23736/S0393-2249.20.03861-8

251. Zaid W, Schlieve T. The Early Effects of Coronavirus Disease-2019 on Head and Neck Oncology and Microvascular Reconstruction Practice: A National Survey of Oral and Maxillofacial Surgeons Enrolled in the Head and Neck Special Interest Group. *J Oral Maxillofac Surg*. Jul 17 2020;doi:10.1016/j.joms.2020.07.012

252. World Health Organization. Pneumonia of unknown cause - China. Updated 5 January 2020. Accessed 28 July, 2020. <https://www.who.int/csr/don/05-january-2020-pneumonia-of-unkown-cause-china/en/>

253. Royal Australasian College of Surgeons. Guidelines for safe surgery: open versus laparoscopic. Updated 9 April 2020. Accessed 26 September, 2020. <https://www.surgeons.org/-/media/Project/RACS/surgeons-org/files/news/covid19-information-hub/2020-04-15-recommendations-on-safe-surgery-laparoscopic-vs-open.pdf?rev=be7927abd03a4a9e976ba944c7c4279a&hash=4E3579010C47C9324C1DB8F8E82F89CD>

254. Royal Australasian College of Surgeons. Guidelines for Personal Protective Equipment. Updated 5 May 2020. Accessed 26 September, 2020. <https://www.surgeons.org/-/media/Project/RACS/surgeons-org/files/news/covid19-information-hub/2020-05-05-covid19-ppe-guidelines.pdf?rev=3a25d9e9f4c444ac8c398b7d7cc7f00a&hash=05156A2797FB43C4AE4F94354C94528B>

255. Royal Australasian College of Surgeons. Surgery triage: responding to the COVID-19 pandemic. Updated 5 May 2020. Accessed 26 September, 2020. <https://www.surgeons.org/-/media/Project/RACS/surgeons-org/files/news/covid19-information-hub/2020-04-22_racs-triage-of-surgery-web.pdf?rev=c28712668d7f45f19ca9df53b77011ea&hash=82A77A9AD9B8A5E23807E449386B80E1>
